# *APLP2* Regulates Refractive Error and Myopia Development in Mice and Humans

**DOI:** 10.1371/journal.pgen.1005432

**Published:** 2015-08-27

**Authors:** Andrei V. Tkatchenko, Tatiana V. Tkatchenko, Jeremy A. Guggenheim, Virginie J. M. Verhoeven, Pirro G. Hysi, Robert Wojciechowski, Pawan Kumar Singh, Ashok Kumar, Gopal Thinakaran, Consortium for Refractive Error and Myopia (CREAM), Cathy Williams

**Affiliations:** 1 Department of Ophthalmology, Columbia University, New York, New York, United States of America; 2 Department of Pathology and Cell Biology, Columbia University, New York, New York, United States of America; 3 School of Optometry & Vision Sciences, Cardiff University, Cardiff, United Kingdom; 4 Department of Ophthalmology, Erasmus Medical Center, Rotterdam, Netherlands; 5 Department of Epidemiology, Erasmus Medical Center, Rotterdam, Netherlands; 6 Department of Twin Research and Genetic Epidemiology, King’s College London School of Medicine, London, United Kingdom; 7 Department of Epidemiology, Johns Hopkins Bloomberg School of Public Health, Baltimore, Maryland, United States of America; 8 Statistical Genetics Section, Inherited Disease Research Branch, National Human Genome Research Institute (NIH), Baltimore, Maryland, United States of America; 9 Department of Ophthalmology, Wayne State University, Detroit, Michigan, United States of America; 10 Department of Anatomy and Cell Biology, Wayne State University, Detroit, Michigan, United States of America; 11 Departments of Neurobiology, Neurology, and Pathology, University of Chicago, Chicago, Illinois, United States of America; 12 School of Social and Community Medicine, University of Bristol, Bristol, United Kingdom; Stanford University School of Medicine, UNITED STATES

## Abstract

Myopia is the most common vision disorder and the leading cause of visual impairment worldwide. However, gene variants identified to date explain less than 10% of the variance in refractive error, leaving the majority of heritability unexplained (“missing heritability”). Previously, we reported that expression of *APLP2* was strongly associated with myopia in a primate model. Here, we found that low-frequency variants near the 5’-end of *APLP2* were associated with refractive error in a prospective UK birth cohort (n = 3,819 children; top SNP rs188663068, p = 5.0 × 10^−4^) and a CREAM consortium panel (n = 45,756 adults; top SNP rs7127037, p = 6.6 × 10^−3^). These variants showed evidence of differential effect on childhood longitudinal refractive error trajectories depending on time spent reading (gene x time spent reading x age interaction, p = 4.0 × 10^−3^). Furthermore, *Aplp2* knockout mice developed high degrees of hyperopia (+11.5 ± 2.2 D, p < 1.0 × 10^−4^) compared to both heterozygous (-0.8 ± 2.0 D, p < 1.0 × 10^−4^) and wild-type (+0.3 ± 2.2 D, p < 1.0 × 10^−4^) littermates and exhibited a dose-dependent reduction in susceptibility to environmentally induced myopia (F(2, 33) = 191.0, p < 1.0 × 10^−4^). This phenotype was associated with reduced contrast sensitivity (F(12, 120) = 3.6, p = 1.5 × 10^−4^) and changes in the electrophysiological properties of retinal amacrine cells, which expressed *Aplp2*. This work identifies *APLP2* as one of the “missing” myopia genes, demonstrating the importance of a low-frequency gene variant in the development of human myopia. It also demonstrates an important role for *APLP2* in refractive development in mice and humans, suggesting a high level of evolutionary conservation of the signaling pathways underlying refractive eye development.

## Author Summary

Gene variants identified by GWAS studies to date explain only a small fraction of myopia cases because myopia represents a complex disorder thought to be controlled by dozens or even hundreds of genes. The majority of genetic variants underlying myopia seems to be of small effect and/or low frequency, which makes them difficult to identify using classical genetic approaches, such as GWAS, alone. Here, we combined gene expression profiling in a monkey model of myopia, human GWAS, and a gene-targeted mouse model of myopia to identify one of the “missing” myopia genes, *APLP2*. We found that a low-frequency risk allele of *APLP2* confers susceptibility to myopia only in children exposed to large amounts of daily reading, thus, providing an experimental example of the long-hypothesized gene-environment interaction between nearwork and genes underlying myopia. Functional analysis of *APLP2* using an *APLP2* knockout mouse model confirmed functional significance of *APLP2* in refractive development and implicated a potential role of synaptic transmission at the level of glycinergic amacrine cells of the retina for the development of myopia. Furthermore, mouse studies revealed that lack of *Aplp2* has a dose-dependent suppressive effect on susceptibility to form-deprivation myopia, providing a potential gene-specific target for therapeutic intervention to treat myopia.

## Introduction

Postnatal refractive eye development is a tightly coordinated process whereby visual experience fine-tunes a genetic program of ocular growth towards an optimal match between the optical power of the eye and its axial length in a process called “emmetropization” [1,2]. The emmetropization process is regulated by a vision-driven feedback loop in the retina and downstream signaling cascades in other ocular tissues, and normally results in sharp vision (emmetropia). Failure to achieve or maintain emmetropia leads to the development of refractive errors, i.e., farsightedness (hyperopia) or nearsightedness (myopia). Myopia is the most common vision disorder worldwide [3]. The prevalence of myopia has increased from 25% to 44% of the adult population in the United States in the last 30 years [4], and reached more than 80% of young adults in some parts of Asia [5,6]. Myopia negatively affects self-perception, job/activity choices, and ocular health [7–9]. Epidemiological data suggest that common myopia represents a major risk factor for a number of potentially blinding ocular diseases such as cataract, glaucoma, retinal detachment, and myopic maculopathy, which is comparable to the risks associated with hypertension for stroke and myocardial infarction, and represents one of the leading causes of blindness [10–12]. It is estimated that 2.5 billion people (1/3 of the world’s population) will be affected by myopia by 2020 [13]. Uncorrected refractive errors are the major cause of vision loss and refractive errors and is one of five World Health Organization’s designated priority health conditions [3,13].

Refractive eye development is controlled by both environmental and genetic factors [14–17]. However, genetic factors are believed to play a key role in determining the impact of environmental factors on refractive eye development, including populations that have experienced rapid rises in the prevalence of myopia in recent decades [14–18]. Human population studies suggest that contribution of genetic factors accounts for 60%-90% of variance in refraction [19–24]. Human genetic mapping studies have identified over 24 chromosomal loci linked to myopia [15,25–30]. However, the currently-identified variants account for only a small fraction of myopia cases [31] suggesting the existence of a large number of yet unidentified low-frequency or small-effect variants, which underlie the majority of myopia cases [32–35]. Here, we present genetic and functional evidence identifying amyloid beta (A4) precursor-like protein 2 (*APLP2*), which was previously found to be involved in synaptic plasticity and transmission in the central nervous system [36–52], as one such myopia-susceptibility gene.

## Results

### Genetic variation at the *APLP2* locus is associated with myopia in children and adults

**Fig 1:**
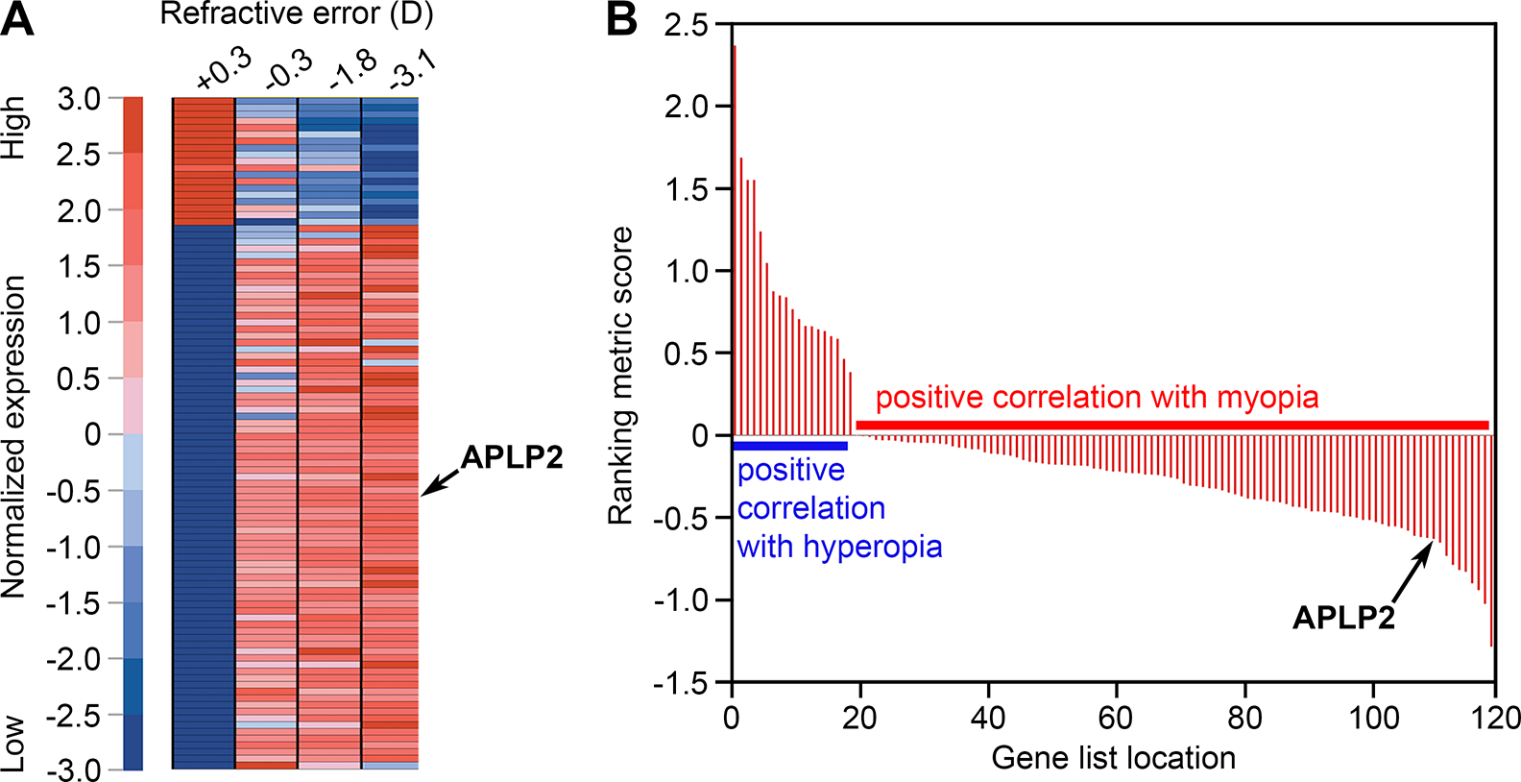
*APLP2* expression is associated with myopic phenotype in the monkey model of myopia. (**A**) Gene set enrichment analysis (GSEA) identified genes differentially expressed in the retina of monkeys with refractive errors induced by form-deprivation. Expression patterns of these genes exhibited statistically significant associations with phenotype “myopia” versus “hyperopia”. The heat map shows genes with the highest positive correlation with either the myopic or hyperopic phenotype. The expression level for each gene was normalized across the samples such that the mean was 0 and the standard deviation (SD) was 3. Expression levels greater than the mean are shaded in red, and those bellow the mean are shaded in blue. The scale (left) indicates SDs above or below the mean. (**B**) Graph showing the distribution of the GSEA correlation (ranking metric) scores for the 119 differentially expressed genes. Ranking metric score reflects the strength of correlation between a gene’s expression pattern and either the myopic or hyperopic phenotype. Positive values indicate a positive correlation with hyperopia and a negative correlation with myopia (i.e., downregulation in myopia and overexpression in hyperopia). Negative values indicate a positive correlation with myopia and a negative correlation with hyperopia (i.e., overexpression in myopia and downregulation in hyperopia). Arrows identify *APLP2*, which was found to be overexpressed in myopia, suppressed in hyperopia, had strong positive association with myopic phenotype and was negatively correlated with hyperopia (ranking metric score -0.63). These analyses were carried out using gene expression data previously reported by Tkatchenko et al. [53].

In a previous study designed to identify genes differentially expressed in myopic eyes, we performed large-scale gene expression profiling in the retina of green monkeys (*Chlorocebus aethiops*) with experimentally induced myopia and identified 119 differentially expressed genes [53]. Here, gene set enrichment analysis (GSEA) [54,55] of these data revealed that expression of one of these genes, *APLP2*, among others was strongly associated with the refractive error phenotype. *APLP2* was found to be overexpressed in myopia and suppressed in hyperopia (Fig 1 and S1 Table).

**Fig 2:**
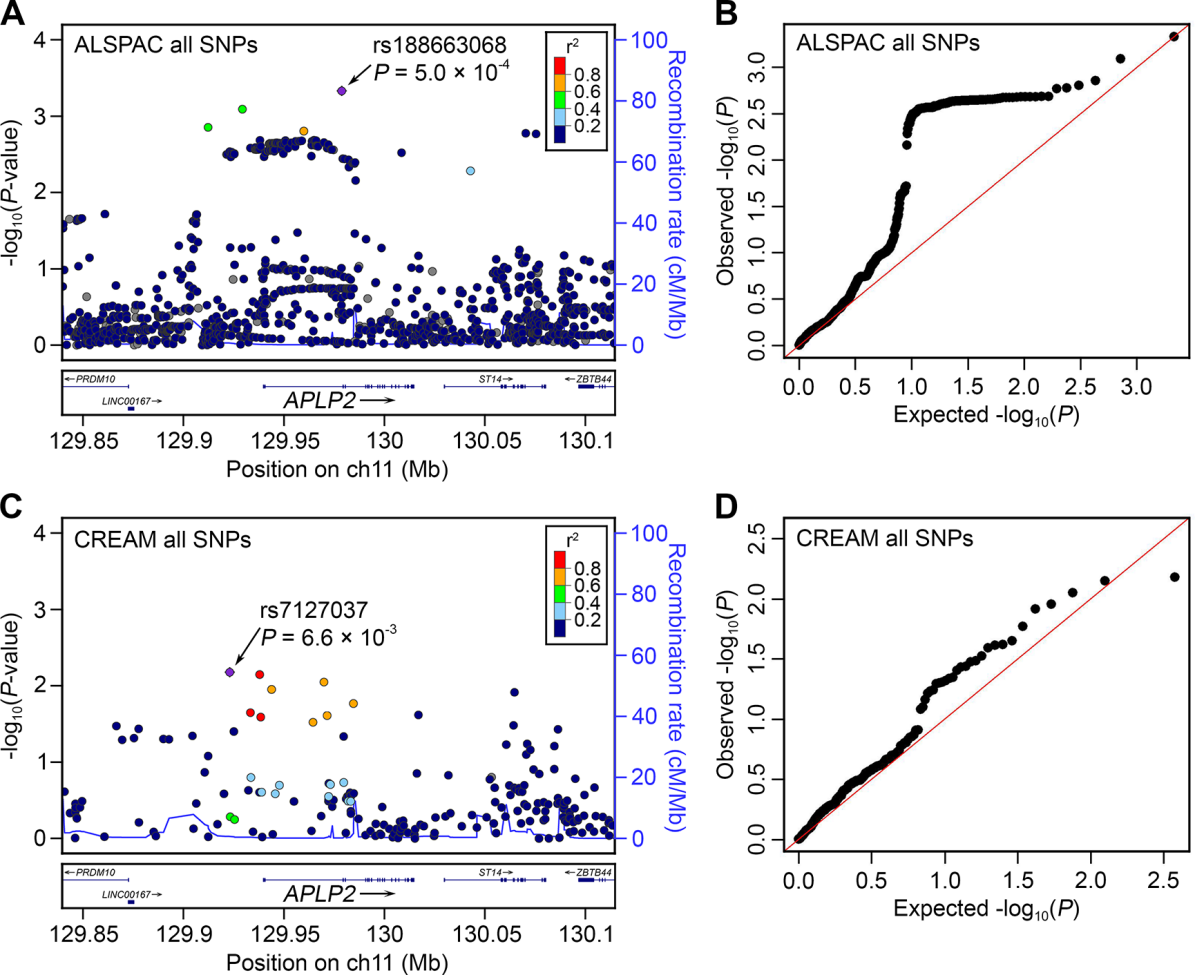
Association between genetic variants at the *APLP2* locus and refractive error in children and adults. The y-axis of all graphs indicates the observed log_10_ (P-values) for single-marker association tests from GWAS for refractive error, for SNPs within 100 kb of the *APLP2* gene in children (n = 3,819) participating in the ALSPAC study (**A, B**) and adults (n = 45,756) participating in the CREAM consortium sample (**C, D**). Region plots for all SNPs examined (**A, C**) show genomic position on the x-axis (build hg19 coordinates) while the colour coding indicates LD (r^2^) with the lead SNP estimated from CEU individuals in HapMap Phase 2, and the right-hand y-axis indicates the recombination rate. Quantile-quantile plots (**B, D**) display expected log_10_ (P-values) on the x-axis.

To explore whether genetic variants within or nearby *APLP2* influence refractive error development in humans, single nucleotide polymorphism (SNP) genetic variants within 100 kb of the *APLP2* gene were tested for association with refractive error in children participating in a UK birth cohort study (the Avon Longitudinal Study of Parents and Children, ALSPAC). Numerous SNPs in an LD block that encompassed the promoter region and 5’-end of the *APLP2* gene were associated with refractive error at age 15 years in ALSPAC participants (Fig 2A and 2B). The most strongly associated variant was rs188663068 (risk allele frequency (RAF) = 0.01, n = 3,819, p = 5.0 × 10^−4^), each copy of the risk allele being associated with a -0.6 D shift in refractive error. Because SNPs in LD do not offer independent evidence of association, permutation testing was used to evaluate whether these results were likely to have arisen by chance. Consistent with the QQ-plot for the full set of SNPs tested (Fig 2B) permutation-based analysis suggested that obtaining a p-value as low as p = 5.0 × 10^−4^ for rs188663068 was not unexpected; however, such an excess of low p-values was unlikely to have occurred by chance (p = 1.4 × 10^−2^). These findings are consistent with the notion that an excess of genetic variants in the promoter region and 5’-end of *APLP2* are associated with refractive error, but because the associated variants have a low minor allele frequency, no single SNP provides compelling evidence on its own. SNPs within 100 kb of the *APLP2* gene were also evaluated in a meta-analyzed refractive error genome-wide association study (GWAS) dataset from the international Consortium for Refractive Error and Myopia (CREAM), which included 45,756 adult individuals from 27 Caucasian and 5 Asian cohorts [30]. As in the ALSPAC cohort, SNPs in the LD block encompassing the promoter region and 5’-end of the *APLP2* gene were most strongly associated with refractive error in the CREAM consortium sample (Fig 2C and 2D; top SNP, rs7127037, p = 6.6 × 10^−3^). The use of permutation testing to account for multiple testing was not possible for the CREAM dataset since we did not have access to the raw genotypes of individual participants. Therefore, as an alternative approach to test for an excess of low p-values in the region found to be associated in the ALSPAC cohort (hg19 chr 11:129904497–129971498), the distribution of p-values inside this region was compared to the surrounding region encompassing 100 kb on the either side of the gene. P-values inside the region were skewed towards low values compared with the p-values outside the region (p = 5.0 × 10^−3^, two-sample Kolmogorov-Smirnov test, S1 Fig).

### Gene-environment interaction between *APLP2* and time spent reading in children with myopia

**Fig 3:**
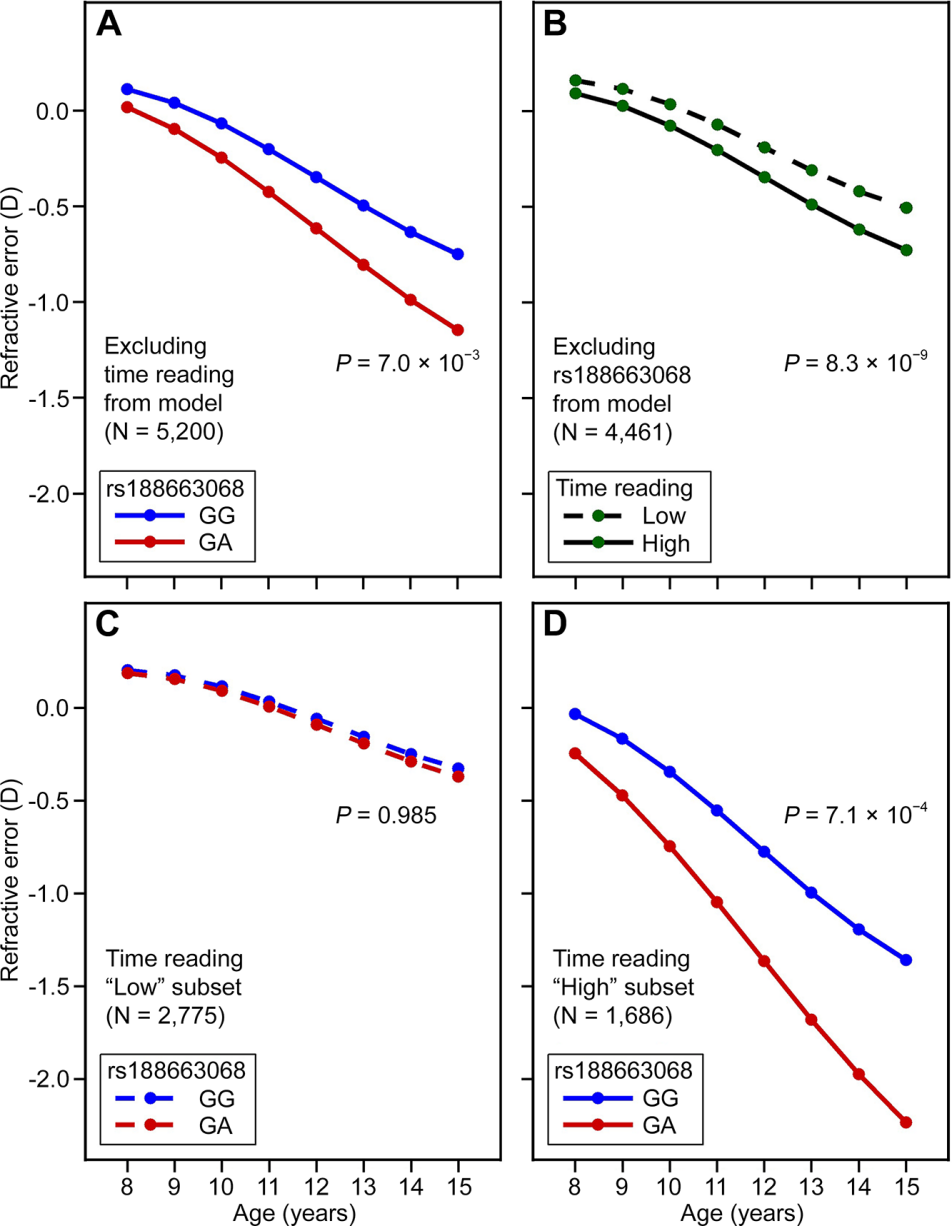
*APLP2* genotype and reading behaviour interact to influence refractive eye development in children. Refractive development in ALSPAC participants (n = 5,200) was modelled over the 8–15 year age range. Models included as a predictor variable either rs188663068 genotype (**A, C, D**) or a binary term categorizing children as spending a “high” or “low” amount of time reading at age 8½ years (**B**). Analyses used the full sample (**A**), those with information available on time spent reading (**B**), or a stratified sample consisting of the low (**C**) or high (**D**) readers.

To explore the possibility of an interaction between *APLP2* gene variants and visual experience, we exploited the availability of longitudinal refractive error measurements over childhood (age range 8 to 15 years) and prospective exposure information regarding the two most important currently known environmental risk factors for myopia, i.e., time spent reading and time spent outdoors. For the strongest *APLP2* risk variant, rs188663068, a “growth trajectory” analysis of refractive development revealed a progressive, age-dependent shift towards a relatively more myopic refractive error in individuals carrying a single copy of the high-risk “A” allele compared to individuals homozygous for the low-risk “G” allele (p = 7.0 × 10^−3^; Fig 3A and S2 Table). When analysed separately, time spent reading ascertained at age 8–9 years, and categorized as “high” or “low”, was also predictive of refractive trajectory in ALSPAC participants, with the “high” reading group also gradually diverging towards a relatively more myopic refractive error as they became older (p = 8.3 × 10^-9^; Fig 3B and S3 Table). A model, which included both rs188663068 and time spent reading as predictors, provided strong evidence for a 3-way interaction between age, time spent reading at age 8–9 years and SNP genotype; implicating gene-environment interaction between the *APLP2* genetic variant and time spent reading that became greater with age (3-way interaction term, p = 4.0 × 10^−3^; S4 Table). Stratifying by time spent reading (“low” versus “high”) revealed that the high-risk “A” allele of rs188663068 was predictive of progression towards myopia only in children who spent a “high” amount of time reading (genotype x age interaction term, p = 0.99 and p = 7.1 × 10^−4^ in “low” and “high” readers respectively; Fig 3C and 3D, S5 and S6 Tables). Logistic regression analysis in the ALSPAC participants at age 15 years confirmed the clinical relevance of the association at the *APLP2* locus (S7 and S8 Tables). The odds ratio (OR) for myopia associated with a single copy of the rs188663068 risk allele was 1.98 (95% CI = 1.02 to 3.87, p = 4.5 × 10^−2^), while by comparison the OR associated with a “high” amount of time spent reading at age 8 years was 1.61 (95% CI = 1.33 to 1.93, p = 6.4 × 10^−7^). Inclusion of an interaction term between rs188663068 genotype and time spent reading supported the presence of an interaction; the OR for myopia of participants in the “high” reading group and carrying a copy of the risk allele was 5.42 (95% CI = 1.15 to 25.52, p = 3.3 × 10^−2^). In linear regression analysis, time spent reading, alone, predicted 0.6% of the variance in refractive error at age 15 years, while the percentage of explained variance increased to 0.9% with the inclusion of rs188663068 genotype and a SNP/reading interaction term (S9 and S10 Tables). There was no evidence for an interaction between rs188663068 genotype and time spent outdoors (S11 and S12 Tables).

### *Aplp2* regulates refractive eye development and susceptibility to myopia in mice

**Fig 4:**
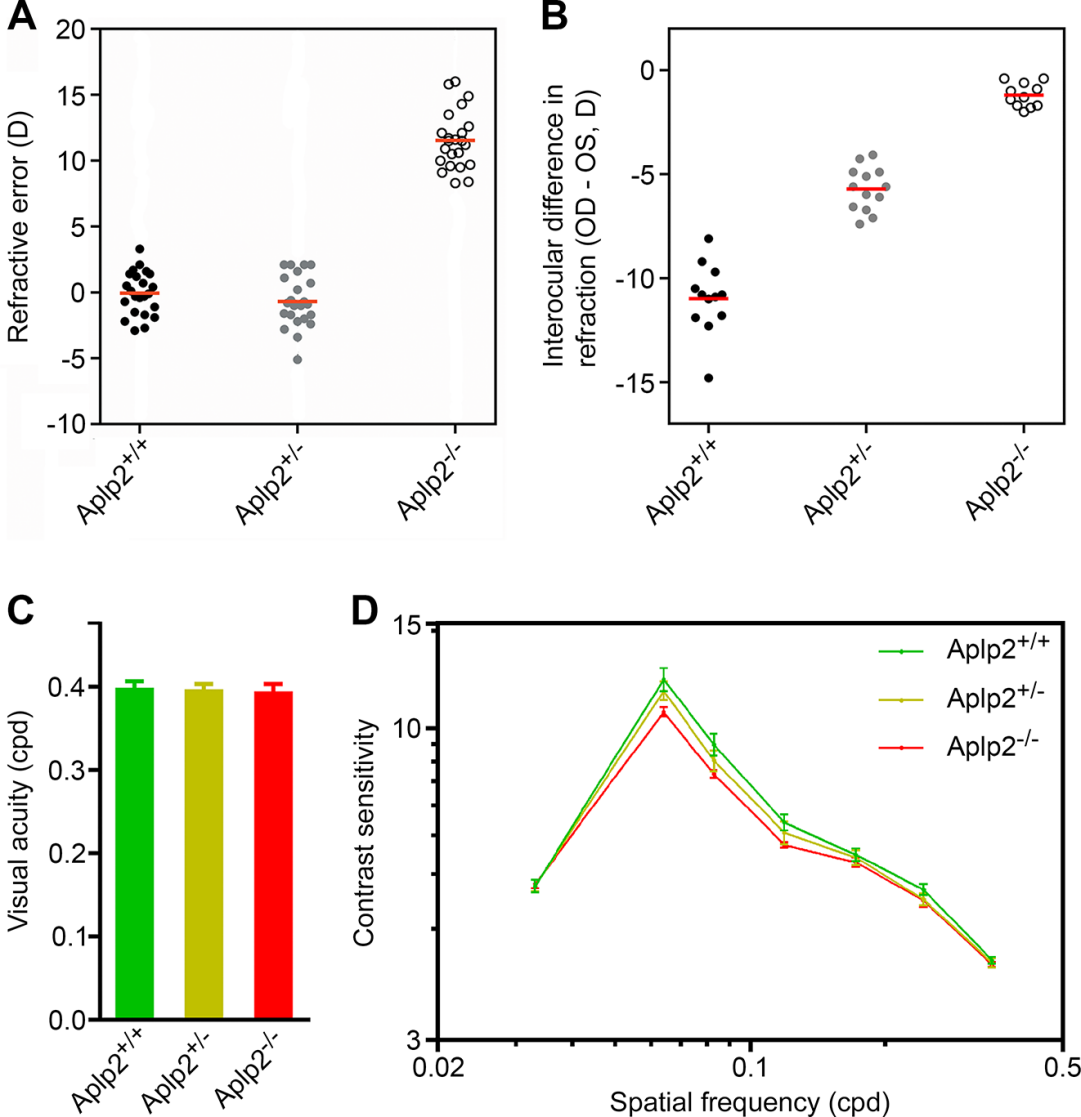
*Aplp2* regulates refractive eye development in the mouse. (**A**) Effect of targeted deletion of *Aplp2* on refractive eye development in the mouse. *Aplp2* knockout mice (generated on C57BL/6J background) develop high degrees of hyperopia (+11.5 ± 2.2 D, p < 1.0 × 10^−4^) compared to both heterozygous (-0.8 ± 2.0 D, p < 1.0 × 10^−4^) and wild-type (+0.3 ± 2.2 D, p < 1.0 × 10^−4^) littermates. Refractive errors were measured at P35 (age when refractive errors stabilize in mice) using automated infrared photorefractor. Red horizontal bars, mean. (**B**) Effect of targeted deletion of *Aplp2* on susceptibility to experimental myopia in mice. Lack of *Aplp2* expression had a negative dose-dependent effect on susceptibility to myopia in mice. Visual form deprivation (VFD) induced -1.2 ± 0.6 D of myopia (p = 3.0 × 10^−2^) in the *Aplp2* knockouts compared to -5.7 ± 1.1 D (p < 1.0 × 10^−4^) in heterozygous and -11.0 ± 1.7 D (p < 1.0 × 10^−4^) in wild-type littermates. VFD was carried out for 21 days from P24 through P45 and refractive status of the deprived eyes versus control eyes was measured using an automated infrared photorefractor (Methods). Red horizontal bars, mean. (**C**) Effect of targeted deletion of *Aplp2* on visual acuity in mice. Visual acuity in *Aplp2* knockouts was not significantly different from that in the heterozygous and wild-type littermates (F(2, 20) = 0.6, p = 0.58). Error bars, s.d.; n = 13. (**D**) Effect of targeted deletion of *Aplp2* on contrast sensitivity in mice. Lack of *Aplp2* resulted in a dose-dependent reduction in contrast sensitivity (F(12, 120) = 3.6, p = 1.5 × 10^−4^). Error bars, s.d.; n = 13. Both visual acuity and contrast sensitivity were measured at P80 using a mouse virtual optomotor system.

To examine whether *APLP2* is functionally involved in refractive error development, we studied refractive eye development in *Aplp2* knockout mice (Fig 4). Mice homozygous for a null allele of the *Aplp2* gene (*Aplp2* ^-/-^ mice) were found to develop high degrees of hyperopia (+11.5 ± 2.2 D, p < 1.0 × 10^−4^) compared to both heterozygous (*Aplp2* ^+/-^) (-0.8 ± 2.0 D, p < 1.0 × 10^−4^) and wild-type (*Aplp2* ^+/+^) (+0.3 ± 2.2 D, p < 1.0 × 10^−4^) littermates (Fig 4A), consistent with the finding that *APLP2* expression is suppressed in hyperopia in monkeys (Fig 1). Visual form deprivation induced -1.2 ± 0.6 D of myopia (p = 3.0 × 10^−2^) in *Aplp2* ^*-/-*^ mice compared to -5.7 ± 1.1 D (p < 1.0 × 10^−4^) in *Aplp2* ^*+/-*^ heterozygotes and -11.0 ± 1.7 D (p < 1.0 × 10^−4^) in wild-type littermates, indicating that lack of *Aplp2* expression has a dose-dependent inhibitory effect on susceptibility to environmentally induced myopia (F(2, 33) = 191.0, p < 1.0 × 10^−4^) (Fig 4B), thus confirming gene-environment interaction between *APLP2* and visual experience identified by human studies.

### *Aplp2* regulates refractive eye development by modulating the function of glycinergic amacrine cells of the retina

**Fig 5:**
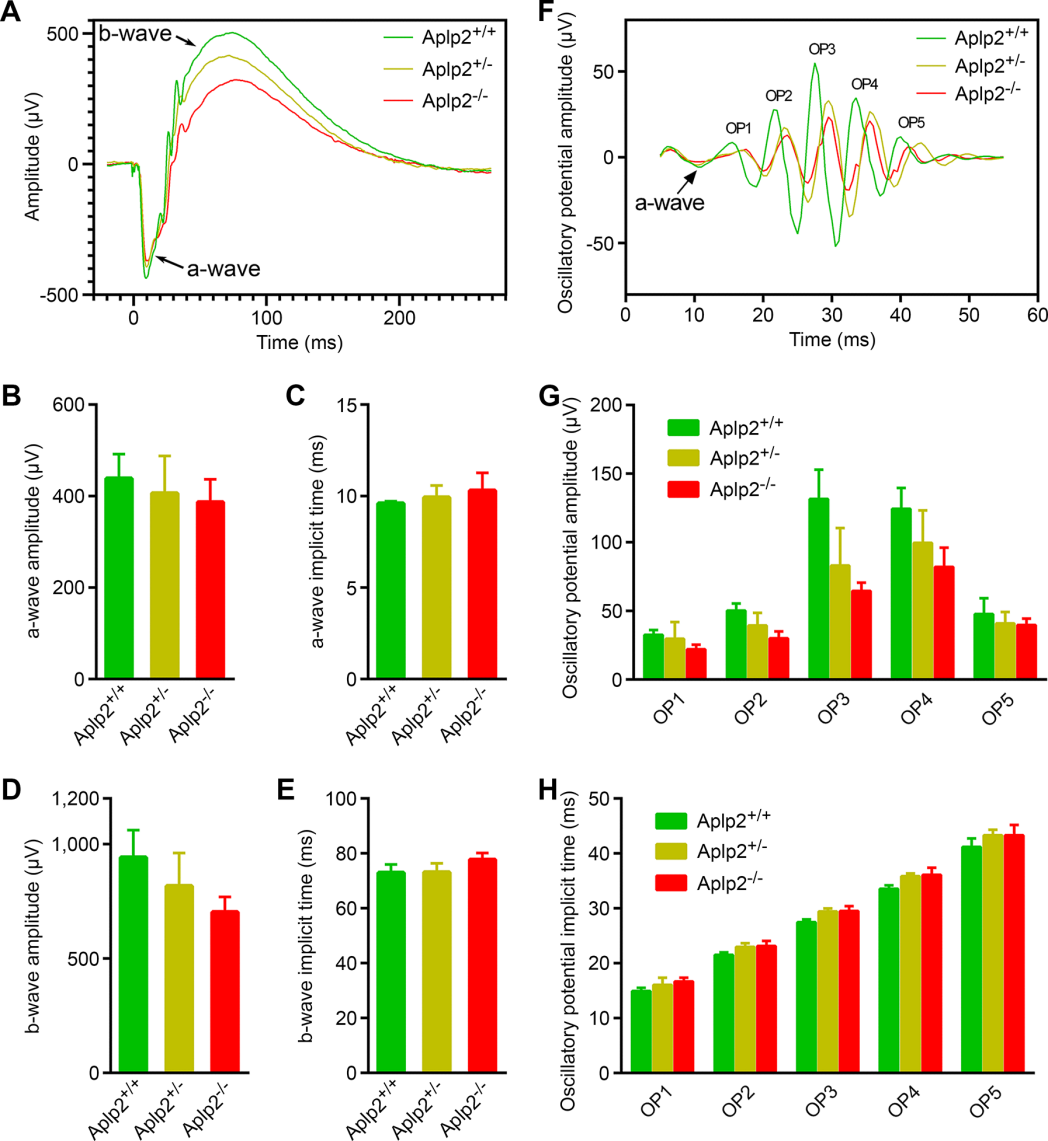
Analysis of scotopic electroretinograms in the *Aplp2* knockout mice. (**A-E**) Effect of targeted deletion of *Aplp2* on the a-wave and b-wave. Lack of *Aplp2* causes a dose-dependent decrease in the b-wave amplitude (F(2, 18) = 6.9, p = 6.0 × 10^−3^). The b-wave implicit time was increased in the *Aplp2* knockouts compared to both heterozygous and wild-type littermates (F(2, 18) = 6.1, p = 9.6 × 10^−3^). Lack of *Aplp2* did not have significant impact on either a-wave amplitude or a-wave implicit time (F(2, 18) = 0.8, p = 0.47, amplitude; F(2, 18) = 2.6, p = 0.1, implicit time). (**F-H**) Effect of targeted deletion of *Aplp2* on oscillatory potentials. The amplitude of the oscillatory potentials (OP) exhibited a dose-dependent decrease in the *Aplp2* knockout mice, while the OP implicit time was increased in both heterozygous and knockout animals compared to the wild-type littermates. OP1 amplitude: F(2, 18) = 3.6, p = 5.0 × 10^−2^; OP2 amplitude: F(2, 18) = 15.6, p = 1.0 × 10^−4^; OP3 amplitude: F(2, 18) = 20.5, p < 1.0 × 10^−4^; OP4 amplitude: F(2, 18) = 9.7, p = 1.0 × 10^−3^; OP5 amplitude: F(2, 18) = 1.9, p = 0.2; OP1 implicit time: F(2, 18) = 7.2, p = 5.0 × 10^−3^; OP2 implicit time: F(2, 18) = 10.9, p = 8.0 × 10^−4^; OP3 implicit time: F(2, 18) = 20.9, p < 1.0 × 10^−4^; OP4 implicit time: F(2, 18) = 17.7, p < 1.0 × 10^−4^; OP5 implicit time: F(2, 18) = 4.5, p = 3.0 × 10^−2^. Error bars, s.d.; n = 7.

**Fig 6:**
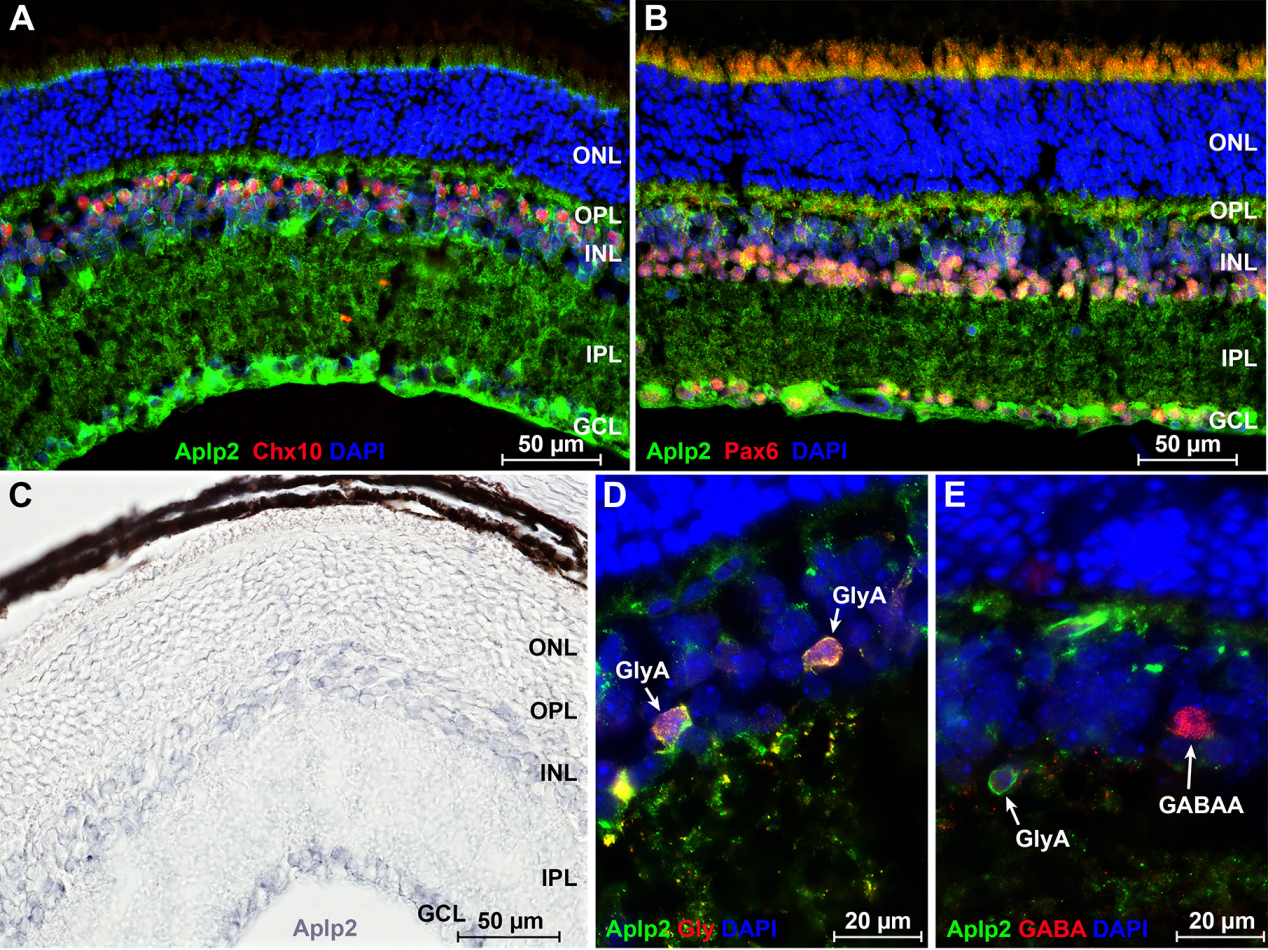
Analysis of *Aplp2* expression in the retina. (**A**) Double staining with antibodies to Chx10 (red), which label bipolar cells, and Aplp2 (green) demonstrate that *Aplp2* is expressed in the bipolar cells of the retina. (**B**) Double staining with antibodies to Pax6 (red), which label amacrine cells, and Aplp2 (green) demonstrate that Aplp2 is expressed in the amacrine cells of the retina. Expression of *Aplp2* is also observed in the ganglion cell layer. (**C**) Analysis of *Aplp2* expression in the retina at the mRNA level using *in situ* hybridization. *In situ* revealed that *Aplp2* is expressed in the inner nuclear and ganglion cell layers of the retina. (**D**) Double staining with antibodies to glycine (red) and Aplp2 (green) revealed that *Aplp2* is strongly expressed in the glycinergic amacrines. Arrows show two glycinergic amacrines with high levels of *Aplp2* expression. (**E**) Double staining with antibodies to GABA (red) and Aplp2 (green) demonstrated that *Aplp2* is not expressed in the GABAergic amacrines. Arrows show a glycinergic amacrine with strong expression of *Aplp2* and an Aplp2-negative GABAergic amacrine. Blue, cell nuclei counterstained with DAPI. GABA, gamma-Aminobutyric acid; GABAA, GABAergic amacrine; GCL, ganglion cell layer; Gly, glycine; GlyA, glycinergic amacrine; INL, inner nuclear layer; IPL, inner plexiform layer; ONL, outer nuclear layer; OPL, outer plexiform layer.

Visual acuity in *Aplp2* ^*-/-*^ mice was not different from that in heterozygous and wild-type littermates (F(2, 20) = 0.6, p = 0.58) (Fig 4C and S13 Table), whereas contrast sensitivity was reduced compared to both heterozygous and wild-type mice (F(12, 120) = 3.6, p = 1.5 × 10^−4^) (Fig 4D and S13 Table). Analysis of scotopic ERGs in *Aplp2* knockouts revealed that lack of *Aplp2* caused a dose-dependent decrease in the amplitude of the b-wave (F(2, 18) = 6.9, p = 6.0 × 10^−3^) (Fig 5A and 5B and 5D) and oscillatory potentials (F(2, 18) = 3.6–20.5, p < 1.0 × 10^−3^) (Fig 5F and 5G); as well as an increase in the implicit time of the b-wave (F(2, 18) = 6.1, p = 9.6 × 10^−3^) (Fig 5C and 5E) and oscillatory potentials (F(2, 18) = 4.5–20.9, p < 5.0 × 10^−3^) (Fig 5F and 5H). Considering that oscillatory potentials are primarily generated by retinal amacrine cells [56,57], which also modulate the amplitude of the b-wave generated by the bipolar cells [58–66], the ERG data suggested that *Aplp2* modulates the function of amacrine cells. Therefore, we then analyzed the expression of *Aplp2* in the retina at the mRNA and protein levels. *In situ* hybridization and immunohistochemical analysis confirmed *Aplp2* expression in both bipolar and amacrine cells of wild-type mice (Fig 6A–6C and S2 Fig). Further analysis also revealed that *Aplp2* was expressed in glycinergic amacrine cells, but was not expressed in GABAergic amacrines (Figs 6D and 6E and S2).

## Discussion

More than 24 chromosomal loci associated with human myopia have been identified [15,25,26,29–31,67–89] either by linkage analysis, which primarily focuses on rare variants with large effect size causing Mendelian forms of myopia, or by large-scale GWAS studies, targeting common variants with moderate effect sizes underlying common myopia. However, refractive error is inherited as a complex quantitative trait thought to be influenced by multiple interacting genes and controlled by dozens and even hundreds of chromosomal loci [19,90–92]. The variants identified to date account for less than 10% of common myopia cases [31], suggesting the existence of a large number of yet unidentified low-frequency and/or small-effect variants, which underlie the majority of myopia cases [32–35].

Several approaches for finding the “missing heritability” of complex traits have been proposed (e.g., increasing GWAS sample sizes, using larger catalogues of human variation, including copy number variations in analyses etc.); however, the most promising route for identification of missing low-frequency and small-effect variants lies through combining biological functional evidence with statistical genetic evidence [33].

Here, we used such “systems genetics” approach, combining gene expression profiling in an animal model of myopia, statistical evidence for association with myopia from two GWAS studies, and functional evidence from a gene-targeted mouse model, to identify *APLP2* as one of the “missing” myopia genes. *APLP2* was first found to be differentially expressed in the retina of monkeys with experimentally-induced myopia. We then found that numerous SNPs within the 5’-end of *APLP2* were associated with refractive error development in children and adults. Furthermore, *Aplp2* also strongly influenced refractive eye development and myopia susceptibility in the gene-targeted mouse model of myopia. Interestingly, *APLP2* is also localized within a broad suggestive myopia locus with LOD score 3.2 identified by Hammond et al. on chromosome 11q23-24 [93].

*APLP2* was first identified as a homologue of the amyloid beta (A4) precursor protein (*APP*) [50] and was assigned to the human chromosome 11q23-25 [49] and the proximal region of mouse chromosome 9 [47]. It was also found that the expression pattern of *APLP2* resembles that of *APP* in the brain and throughout the body, with particularly high expression in neurons of the central and peripheral nervous system [50,94]. The biological role of *APLP2* was investigated using gene-targeted mouse mutants. *Aplp2* knockout mice were normal in size, fertile, and appeared healthy, whereas 80% of *Aplp2*/*APP* double knockout animals died within 24 h after birth and the remaining 20% exhibited difficulty in righting, ataxia, spinning behavior, and a head tilt [45,52,95]. This was contrasted by no lethality or apparent abnormalities in *Aplp1*/*APP* double knockouts, suggesting that the *Aplp2* plays a key role in neuronal development and function [52,95–97]. In the brain, APLP2 protein has been localized to the presynaptic active zone of neuronal axons in close proximity to the synaptic vesicles [36]. Consistent with these data, it has been reported that the lack of an Aplp2/APP complex results in reduced expression of vesicular glutamate transporter 2 (*VGLUT2*) and a defect in synaptic transmission [40,41], as well as reduced spatial learning and synaptic plasticity [37–39]. These effects of *Aplp2* on neuronal function have been suggested to be mediated by its role in the subtle modulation of neurotransmitter release [36,39]. Our observation in mice that *APLP2* modulates the electrophysiological properties of the retina is consistent with its role in synaptic transmission. We found that lack of *Aplp2* led to a significant dose-dependent suppression of the b-wave and oscillatory potentials of the ERG. Considering that oscillatory potentials are primarily generated by the amacrine cells [56,57], which also modulate the amplitude of the b-wave generated by the bipolar cells [58–66], the ERG data suggest that *Aplp2* influences refractive eye development by modulating the function of amacrine cells. Interestingly, this is consistent with a previously suggested role for amacrine cells in the regulation of refractive eye development [98–108]. The involvement of *Aplp2* in the regulation of retinal processing at the level of amacrine cells is further corroborated by our findings that *Aplp2* is expressed in the glycinergic amacrine cells of the retina, which provide feed-forward and feedback inhibition in the retina and play important role in contrast processing [109–115].

Retinal blur associated with inaccurate accommodation during nearwork (so called accommodative lag) and peripheral hyperopic defocus have been hypothesized to be the driving force behind myopia progression in children [116–118]. We found that *Aplp2* modulated sensitivity to retinal image degradation induced by form-deprivation in a dose-dependent manner, yet, lack of *Aplp2* did not cause an observable change in visual acuity. Thus, our data, in conjunction with the published data on the role of *APLP2* in neuronal function, suggest that *APLP2* most likely modulates sensitivity to the degradation of retinal images by regulating the processing of contrast by the retina via modulation of synaptic transmission at the level of glycinergic amacrines. The reduced susceptibility to myopia in mice lacking *Aplp2* makes lowering the level of APLP2 in the retina via gene therapy an appealing future direction for therapeutic intervention in human myopia.

In summary, we have identified *APLP2* as a novel gene involved in refractive eye development and associated with human myopia. The role of *APLP2* in human myopia is supported by several lines of evidence, which suggest that genetic variation at the *APLP2* promoter region may influence *APLP2* expression in the inner retina and, in turn, may modulate synaptic transmission at the level of amacrine cells, leading to alterations in refractive development. Consistent with its important role in neuronal development and function, *APLP2* appears to have been subject to intense evolutionary pressure evidenced by 97.7% DNA sequence conservation of the gene between humans and mice. Our findings that naturally occurring genetic variation at the *APLP2* locus was associated with myopia only in children who spent an above-average time reading and observations of an analogous gene-environment interaction between *Aplp2* and visual input in mice also imply a high level of evolutionary conservation for the pathways underlying refractive eye development. Further work will be required to pinpoint the causal variant(s) at the *APLP2* locus that determine susceptibility to myopia, and to elucidate whether (as suggested by the location of the most strongly associated variant in the promoter region of the gene) they alter the level of *APLP2* expression. Future functional studies will also need to explore the role of *APLP2* in synaptic transmission at the level of amacrine cells and its role in defocus processing.

## Materials and Methods

### Tests for association with genetic variants at the *APLP2* locus in children

To investigate whether naturally-occurring genetic variation at the *APLP2* locus influences refractive development in children, data from an existing British birth cohort were examined. The Avon Longitudinal Study of Parents and Children (ALSPAC) recruited 14,541 pregnant women resident in Avon, UK with expected dates of delivery 1st April 1991 to 31st December 1992. Of the initial 14,541 pregnancies, 13,988 children were alive at 1 year. Data collected included self-completion questionnaires sent to the mother, to her partner and after age 5 to the child; direct assessments and interviews in a research clinic; biological samples and linkage to school and hospital records. The original cohort was largely representative of the UK 1991 Census; however, there was trend for greater loss at follow-up for families of low socioeconomic status and of non-White ethnic origin [119]. Ethical approval for the study was obtained from the ALSPAC Law and Ethics committee and the three local research-ethics committees.

Refractive error was assessed using non-cycloplegic autorefraction at research clinics attended when children were approximately 7½, 10½, 11½, 12½ and 15½ years of age [120]. DNA samples from the participants were genotyped on Illumina HumanHap 550 bead arrays [121]. Data were available for a total of 464,311 autosomal SNPs that passed quality control filters [121] in 8,365 individuals of European ancestry (as demonstrated by clustering with HapMap CEU individuals upon multidimensional scaling analysis). SNPs at non-genotyped loci were imputed with MACH,[122] using the 1000-genomes project GIANT consortium November 2010 data release as a reference panel. For attendees at the 15½ year clinic (n = 3,819), association between non-cycloplegic refractive error and imputed genotype dosage was tested using mach2qtl for SNPs within 100 kb of the *APLP2* gene. Age and sex were included as covariates in the analysis. Permutation testing was used to generate empirical p-values that accounted for multiple testing and for LD between markers. It was carried out by assigning subjects a new phenotype, sampled randomly without replacement from the true list of phenotypes, and repeating the tests for association between the new trait and the genotype for all SNPs in the region. From 1000 such permutations, the probability of observing a p-value as low as that found in the real dataset was estimated. To test for an excess of low p-values, the probability of observing the 5th percentile p-value from the real dataset was estimated from the 5th percentile p-values observed in the 1000 permutations.

To explore whether the most strongly associated SNP at the *APLP2* locus, rs188663068, acted early or late in childhood, the imputed genotype for this SNP was included as a fixed effect term in a linear mixed model of childhood refractive error “trajectory” in ALSPAC participants (note that because of the very low risk allele frequency (RAF) of rs188663068, the single subject who was homozygous for the risk allele (genotype AA) was re-coded as a heterozygote (GA). The model also included sex, age, age^2^ and age^3^ as fixed effects, while refractive error over the age range 7½ to 15½ years and a linear age term were modelled as random effects. Data were included for 5,200 ALSPAC participants for whom non-cycloplegic autorefraction readings had been obtained on at least 3 occasions (specifically, there were 833 subjects with data available from 3 visits, 1,696 with data from 4 visits, and 2,671 with data from all 5 visits). Linear mixed modelling was performed using the *lme* function in R.

More complex refraction trajectory models were constructed by including additional predictor variables. Time spent reading was ascertained from a questionnaire completed by the mother when the ALSPAC participants were aged approximately 8½ years as previously described [120]. In response to the question “On normal days in school holidays, how much time on average does your child spend each day reading books for pleasure”, children were classified as either spending a “high” (response “1–2 hours” or “3 or more hours” per day) or “low” (response “None at all” or “1 hour or less”) amount of time reading. The time reading variable was coded as “low” = 0 (reference) and “high” = 1. There were 2,775 and 1,686 subjects in the “low” and “high” subsets, respectively (note that information on time spent reading was missing for 739 of the 5,200 participants in the refraction trajectory sample). Although time spent reading was sampled at only a single age-point, reading behaviour may track forward as children get older. Therefore, the time spent reading variable’s predictive capacity may stem from capturing inter-subject variation not only at the age of 8–9 years, but also to some extent inter-subject variation in reading behaviour at older ages. Time spent outdoors was gauged from a separate item on the same questionnaire: “On a school weekday, how much time on average does your child spend each day out of doors in summer?”. Children were classified as spending a “high” amount of time outdoors if the response was “1–2 hours” or “3 or more hours”, and as “low” otherwise. Note that this questionnaire response was selected for the present study instead of a closely-related one used previously [120], since the former provided an approximately equal split of the sample, while the latter variable resulted in an ~1:9 ratio of subjects classified as spending a low versus high amount of time outdoors, and thus despite its slightly greater predictive discrimination of incident myopia, it would have led to very small numbers of subjects in the “low time outdoors + GA rs188663068 genotype” group. The time outdoors variable was coded as “low” = 0 (reference, n = 2,349) and “high” = 1 (n = 2,145) and there were 706 children with missing information. Sex was not significantly associated with refractive error in the refraction trajectory analyses and so was dropped from the models.

To confirm the refraction trajectory results, association between rs188663068 genotype (coded GG = 0, GA = 1) and refractive error was also analyzed using linear and logistic regression for subjects attending the ALSPAC research clinic; targeting the children when aged 15 years. All children with information available were included in these models to maximise precision of risk estimates. Time spent reading and time spent outdoors were included, separately, as predictors, using the coding scheme described above, in models with and without an interaction term (rs188663068 genotype x time reading, etc.). Sex was not significantly associated with refractive error in these linear regression analyses and so was dropped from the models. Age-at-baseline was not included since, being a birth cohort, the age interval was narrow at each target age. As in the refraction trajectory analyses, the single subject with rs188663068 genotype AA was re-coded as GA.

### Tests for association with genetic variants at the *APLP2* locus in adults

Genetic variants at the *APLP2* locus were examined using meta-analyzed data from the international genome-wide association study (GWAS) of refractive error carried out by the Consortium for Refractive Error and Myopia (CREAM) [30]. The CREAM meta-analysis included data from 32 studies: 1958 British Birth Cohort, ALSPAC (mothers), ANZRAG, AREDS1a1b, AREDS1c, Beijing Eye Study, BMES, CROATIA-Korcula, CROATIA-Split, CROATIA-Vis, DCCT, EGCUT, ERF, FECD, FITSA, Framingham, GHS 1, GHS 2, KORA, OGP Talana, ORCADES, RS1, RS2, RS3, SCES, SIMES, SINDI, SP2, TEST/BATS, TwinsUK, WESDR, and YFS. Each study received prior approval from its local medical ethics committee, and written informed consent was obtained from all participants in accordance with the tenets of the Declaration of Helsinki. The age of subjects in each sample ranged from 31.4 to 79.9 years and, apart from 3 samples, contained an approximately equal split of males/females. Twenty-seven samples comprised of subjects of European ancestry, while 5 were of Asian ancestry. GWAS analyses were carried out for spherical equivalent refractive error (dependent variable) with genotype dosage, age and sex included as independent variables, and meta-analysis was done under a random-effects model, as described [30]. SNPs within 100 kb of the *APLP2* gene were evaluated. Permutation-based analysis to correct for multiple testing could not be carried out for the CREAM GWAS dataset since we did not have access to the raw genotypes. Therefore, for SNPs within 100 kb of *APLP2*, the distribution of CREAM p-values inside versus outside the region showing strong association in the ALSPAC sample (hg19 chr 11:129904497–129971498; hg18 chr 11:129409707–129476708) was compared using the two-sample Kolmogorov-Smirnov test.

### *Aplp2* knockout mice

*Aplp2* knockout mice (B6.129S7-Aplp2^tm1Dbo^/J) were obtained from the Jackson Laboratory (Bar Harbor, ME) as heterozygotes and were maintained as an in-house breeding colony on a C57BL/6J background, which was shown not to carry Rd mutations that cause retinal degeneration in mice [123]. To generate homozygous (*Aplp2* ^-/-^), heterozygous (*Aplp2* ^+/-^) and wild-type (*Aplp2* ^+/+^) animals for the experiments, heterozygous males and females were bred and resulting offspring were genotyped as previously described [45] to identify animals of different genotypes. Only littermates were used for all experiments to ensure isogenic genetic background. All animals received water and food ad libitum. All mouse procedures adhered to the ARVO Statement for the Use of Animals in Ophthalmic and Vision Research and were approved by the Columbia University Institutional Animal Care and Use Committee (Protocol #AAAK2700). Animals were anesthetized via intraperitoneal injection of pentobarbital (50 mg/kg), or via intraperitoneal injection of ketamine (90 mg/kg) and xylazine (10 mg/kg). Animals were euthanized by cervical dislocation while under full surgical anesthesia.

### Analysis of refractive eye development in *Aplp2* knockout mice

Visually guided emmetropization normally results in children who are born myopic becoming less myopic and children who are born hyperopic becoming less hyperopic during the early postnatal period [1]. In mice, both the variability and magnitude of the refractive error are reduced during the early postnatal period (P21-P40) indicating emmetropization [124–126]. In C57BL/6J mice, refractive error stabilizes around emmetropia at ~P32. To examine the role of *Aplp2* in emmetropization, we analyzed refractive eye development in mice homozygous (*Aplp2* ^-/-^) and heterozygous (*Aplp2* ^+/-^) for a null allele of the *Aplp2* gene, as well as in the wild-type animals (*Aplp2* ^+/+^). The refractive state of both left and right eyes was determined on alert animals at P21, P35, and P67 using an automated eccentric infrared photorefractor as previously described [124,127]. The animal to be refracted was immobilized using a restraining platform, and each eye was refracted along the optical axis in dim room light (< 1 lux), 20–30 min. after instilling 1% tropicamide ophthalmic solution (Alcon Laboratories, Inc., Fort Worth, TX) to ensure mydriasis and cycloplegia. Five independent measurement series (~300–600 measurements each) were taken for each eye. The measurements were automatically acquired by the photorefractor every 16 msec. Each successful measurement series (i.e., Purkinje image in the center of the pupil and stable refractive error for at least 5 sec.) was marked by a green LED flash, which was registered by the photorefractor software. Sixty individual measurements from each series, immediately preceding the green LED flash, were combined, and a total of 300 measurements (60 measurements x 5 series = 300 measurements) were used to calculate the refractive error mean and standard deviation.

### Analysis of gene-environment interaction between *Aplp2* and visual experience in *Aplp2* knockout mice

Human population studies revealed that environmental factors, such as nearwork and reading, play important role in the development of myopia [128–131]. These findings were complemented by observations that nearwork and reading are associated with the lag of accommodation, i.e., insufficiently strong accommodative response for near objects, which places the plane of best focus behind the retina (producing slight optical blur) when the subject performs nearwork tasks [116,117]. Optical blur produced by the lag of accommodation is the signal that drives excessive eye growth and causes myopia [116,129,132–135]. Animal studies also demonstrated that excessive eye growth and myopia can be induced in species as diverse as the fish, chicken, tree shrew, monkeys, guinea pig and, most recently, mouse by retinal image degradation or optical blur (recapitulated in animal models by placing a diffuser or a negative lens in front of the eye) [136–143].

To examine the role of *Aplp2* in the development of environmentally induced myopia, we analyzed the effect of diffuser-imposed retinal image degradation (visual form deprivation) on refractive eye development in mice homozygous (*Aplp2* ^-/-^) and heterozygous (*Aplp2* ^+/-^) for a null allele of the *Aplp2* gene, as well as in the wild-type mice (*Aplp2* ^+/+^). Visual input was degraded in one of the eyes by applying plastic diffusers, and refractive development of the treated eye was compared to that of the contralateral eye, which was not treated with a diffuser, as previously described [126,143]. Diffusers represented low-pass optical filters, which severely degraded the image projected onto the retina by removing high spatial frequency details. Frosted hemispherical plastic diffusers were hand-made using caps from 0.2 ml PCR tubes (Molecular BioProducts, San Diego, CA) and rings made of medical tape (inner diameter 6 mm; outer diameter 8 mm). A cap was frosted with fine sandpaper and attached to a ring with Loctite Super Glue (Henkel Consumer Adhesives, Avon, OH). On the first day of the experiment (P24), animals were anesthetized via intraperitoneal injection of pentobarbital (50 mg/kg), and diffusers were attached to the skin surrounding the right eye with three stitches using size 5–0 ETHILON microsurgical sutures (Ethicon, Somerville, NJ) and reinforced with Vetbond glue (3M Animal Care Products, St. Paul, MN) (the left eye served as a control). Toenails were covered with adhesive tape to prevent mice from removing the diffusers. Animals recovered on a warming pad and were then housed under low-intensity constant light in transparent plastic cages for the duration of the experiment as previously described [126,143]. Following 21 days of visual form deprivation (from P24 through P45), diffusers were removed and refractive status of both treated and control eyes was assessed using an automated eccentric infrared photorefractor as previously described [144]. The interocular difference in refraction between the treated and contralateral control eye served as an indication of the extent of induced myopia.

### Analysis of visual acuity and contrast sensitivity in *Aplp2* knockout mice

To examine the role of *Aplp2* in the overall visual function, we compared visual acuity and contrast-sensitivity in the *Aplp2* knockout mice (*Aplp2* ^-/-^), mice heterozygous (*Aplp2* ^+/-^) for a null allele of the *Aplp2* gene, and in the wild-type littermates (*Aplp2* ^+/+^). Both visual acuity and contrast sensitivity were measured at P80 using a virtual optomotor system (Mouse OptoMotry System, Cerebral Mechanics, Medicine Hat, AB Canada), as previously described [145]. Briefly, the animal to be tested was placed on a platform surrounded by four computer screens displaying a virtual cylinder comprising a vertical sine wave grating in 3D coordinate space. The OptoMotry software controlled the speed of rotation, direction of rotation, the frequency of the grating and its contrast.

To measure visual acuity, the initial spatial frequency of the grating was set at 0.1 cycles/degree and the contrast was set at maximum. The frequency was then systematically increased using staircase procedure until the maximum spatial frequency capable of eliciting a response (visual acuity) was determined. The staircase procedure was such that 3 correct answers in a row advanced it to a higher spatial frequency, while 1 wrong answer returned it to a lower frequency.

Contrast sensitivity function was measured at seven spatial frequencies, i.e. 0.033, 0.064, 0.083, 0.119, 0.172, 0.244, and 0.347 cycles/degree, using the staircase procedure described above. The contrast sensitivity at each frequency was calculated as a reciprocal of the contrast threshold, which was calculated as a Michelson contrast from the screen luminances ($\frac{I_{\max}\;\text{-}\;I_{\min}}{I_{\max}+I_{\min}}$; I_white_ = 208.25 cd/m^2^, I_black_ = 0.21 cd/m^2^).

### Analysis of electrophysiological properties of the mouse retina

Dark-adapted electroretinograms (ERGs) are particularly sensitive to changes in the inner retina [56]; therefore, to assess the effect of *Aplp2* on the electrophysiological properties of various neuronal populations in the retina, we analyzed scotopic ERGs in the *Aplp2* knockout (*Aplp2* ^-/-^) mice, mice heterozygous for a null allele of the *Aplp2* gene (*Aplp2* ^+/-^ mice), and in the wild-type littermates (*Aplp2* ^+/+^ mice). Animals to be used for ERG were dark-adapted overnight. Prior to ERG recordings, dark-adapted mice were anesthetized via intraperitoneal injection of ketamine (90 mg/kg) and xylazine (10 mg/kg) and placed on a heating pad. The pad was connected to a rectal probe and thermostat via a feedback circuit, which maintained the body temperature at 37°C. Pupil dilation was achieved by instilling one drop of 1% tropicamide ophthalmic solution (Alcon Laboratories, Inc., Fort Worth, TX) in each eye at the time of anesthesia. Silver-embedded thread corneal recording electrodes were positioned across the apex of each cornea anesthetized with Lidocaine and held in place with 2.5% Goniovisc ophthalmic solution (HUB Pharmaceuticals, Rancho Cucamonga, CA) and optically clear mini contact lenses (Ocuscience, Rolla, MO). Stainless steel sub-dermal needle reference electrodes were placed subcutaneously below each eye along the upper jaw, while the ground electrode was inserted into the base of the tail. ERGs were recorded using Ocuscience rodent ERG system (Rolla, MO). To elicit retinal responses, each eye was presented with 5-msec white-light flashes of increasing intensity produced by a mini-Ganzfeld stimulator. Nine intensities ranging from 0.001 cd•s/m^2^ to 32 cd•s/m^2^ were used with the interstimulus interval increasing from 18 sec to 120 sec with the increase in the stimulus intensity (12-sec increase for each step). Responses to three flashes of each intensity were recorded and averaged. ERG data were processed and quantified using ERGVIEW software package (Ocuscience, Rolla, MO).

### Analysis of *Aplp2* expression in the eye

To identify the tissues and cell types in which *Aplp2* is expressed, we examined expression of *Aplp2* in the eye using *in situ* hybridization and immunohistochemistry. *In situ* hybridizations were performed essentially as previously described [146]. Briefly, C57BL/6J mouse eyes were enucleated at P27 and used to prepare eyecups by removing the cornea and lens in ice-cold 1 X PBS. The eyecups were then fixed in 4% paraformaldehyde in 1 X PBS overnight at 4°C, cryoprotected in 30% sucrose in 1 X PBS and embedded in Tissue-Tek O.C.T compound (Sakura Finetek USA, Torrance, CA). 10-μm cryostat sections were incubated with 1 μg/ml Proteinase K in 1 X PBS, washed in 2 mg/ml Glycine in 1 X PBS, incubated with 0.25% acetic anhydride in 0.1 M TEA buffer, and hybridized with digoxigenin(DIG)-labeled cDNA probes followed by incubation with anti-DIG antibodies conjugated with alkaline phosphatase (AP) (Roche Applied Science, Indianapolis, IN). The AP activity was localized and signal was detected using NBT (0.25 mg/ml) and BCIP (0.125 mg/ml) (Roche Applied Science, Indianapolis, IN) as substrates.

For immunohistochemistry, eyecups were prepared as described above, fixed in 2% formaldehyde in 1 X PBS for 4 hours on ice, washed in 1 X PBS, cryoprotected in 30% sucrose in 1 X PBS, and embedded in Tissue-Tek O.C.T compound (Sakura Finetek USA, Torrance, CA). 10-μm cryostat sections were washed with 1 X PBS, blocked with 5% normal goat serum, 5% BSA, 0.1% fish gelatin, 0.1% Triton X-100 and 0.05% Tween 20 in 1 X PBS (blocking buffer) for 1 hour at room temperature, and then incubated with rabbit anti-Aplp2 primary antibodies (D2-II, dilution 1:1,000) [46] in blocking buffer overnight at 4°C. The sections were then washed with 0.2% Triton X-100 in 1 X PBS (PBT) and incubated with Alexa-488-conjugated donkey anti-rabbit secondary antibodies (1:500, Life Technologies, Grand Island, NY) in blocking buffer for 2 hours at room temperature. After sections were washed in PBT, they were incubated with sheep anti-Chx10 (1:200, Abcam, Cambridge, MA), or rabbit anti-Pax6 (1:1,000, Abcam, Cambridge, MA), or rabbit anti-GABA (1:100, EMD Millipore, Billerica, MA), or rabbit anti-Glycine (1:100, EMD Millipore, Billerica, MA) primary antibodies overnight (48 hours for anti-GABA and anti-Glycine antibodies) at 4°C; followed by the washes in PBT and incubation with Alexa-594-conjugated donkey anti-sheep (1:500, Life Technologies, Grand Island, NY) or donkey anti-rabbit (1:500, Life Technologies, Grand Island, NY) secondary antibodies in blocking buffer for 2 hours at room temperature. The slides were then again washed in PBT, incubated with 300 nM DAPI in 1 X PBS, and mounted in ProLong Gold antifade mountant (Life Technologies, Grand Island, NY). The colocalization between Aplp2 and other antigens was examined and image capture was performed using laser scanning confocal microscope Leica TCS SP5 (Leica Microsystems, Buffalo Grove, IL) and the manufacturer’s software.

## Supporting Information

S1 Text The Consortium for Refractive Error and Myopia (CREAM)–membership list. (PDF)

S1 Fig Distribution of p-values inside and outside of the *APLP2* region–CREAM dataset. The distribution of p-values was skewed towards unexpectedly low values for SNPs in the region that showed association in ALSPAC participants (hg19 chr 11:129904497–129971498) compared to the surrounding region. The distribution inside the region was significantly skewed towards lower p-values (p = 0.005; two-sample Kolmogorov-Smirnov test) and significantly different from a uniform distribution (p = 0.001; two-sample Kolmogorov-Smirnov test). (**A**) Schematic diagram of the two regions in which the distribution of p-values was compared: i) the region where a strong association between SNP genotypes and refractive error was observed in the ALSPAC cohort (green shading); and ii) the surrounding region. (**B**) Distribution of p-values for SNPs within the region showing association in ALSPAC cohort. (**C**) Distribution of p-values for SNPs in the surrounding region.
(TIF)

S2 Fig Expression of *Aplp2* in the inner retina–immunohistochemistry. In the inner nuclear layer of the retina, *Aplp2* was expressed in the bipolar cells and glycinergic amacrines. (**Top panel**) Co-localization of Aplp2 and Chx10 demonstrating expression of *Aplp2* in the bipolar cells. Magenta arrows, Aplp2- and Chx10-positive bipolar cells; white arrows, Aplp2-positive Chx10-negative cell. (**Second and third panels**) Co-localization of Aplp2 and Pax6 demonstrating expression of *Aplp2* in the amacrine cells. Magenta arrows, Aplp2-positive Pax6-positive amacrine cells; white arrows, Aplp2-negative Pax6-positive amacrine cells. (**Fourth panel**) Co-localization of Aplp2 and glycine demonstrating expression of *Aplp2* in the glycinergic amacrines. Magenta arrows, Aplp2-positive glycine-positive amacrine cells. (**Bottom panel**) Co-localization of Aplp2 and GABA demonstrating lack of *Aplp2* expression in the GABAergic amacrines. Magenta arrows, Aplp2-negative GABA-positive amacrine cell; white arrows, Aplp2-positive GABA-negative (glycinergic) amacrine cell.
(TIF)

S1 Table Gene set enrichment analysis–correlation with the depth of the vitreous chamber. (DOCX)

S2 Table Refractive error “growth trajectory” analysis in ALSPAC subjects. Model excluding time reading term (n = 5,200).
(DOCX)

S3 Table Refractive error “growth trajectory” analysis in ALSPAC subjects. Model excluding SNP term (n = 4,461).
(DOCX)

S4 Table Refractive error “growth trajectory” analysis in ALSPAC subjects. Full model (n = 4,461).
(DOCX)

S5 Table Refractive error “growth trajectory” analysis in ALSPAC subjects. Model restricted to time reading “Low” subset (n = 2,775).
(DOCX)

S6 Table Refractive error “growth trajectory” analysis in ALSPAC subjects. Model restricted to time reading “High” subset (n = 1,686).
(DOCX)

S7 Table Logistic regression model for myopia at age 15½ years in ALSPAC subjects. Time reading (“Low” versus “High”) (n = 3,312). Without interaction term.
(DOCX)

S8 Table Logistic regression model for myopia at age 15½ years in ALSPAC subjects. Time reading (“Low” versus “High”) (n = 3,312). With interaction term.
(DOCX)

S9 Table Linear regression model for refractive error at age 15½ years in ALSPAC subjects. Time reading (“Low” versus “High”) (n = 3,312).
(DOCX)

S10 Table Linear regression model for refractive error at age 15½ years in ALSPAC subjects. Time reading (“Low” versus “High”) (n = 3,312).
(DOCX)

S11 Table Linear regression model for refractive error at age 15½ years in ALSPAC subjects. Time outdoors (“Low” versus “High”) (n = 3,329).
(DOCX)

S12 Table Linear regression model for refractive error at age 15½ years in ALSPAC subjects. Time outdoors (“Low” versus “High”) (n = 3,329).
(DOCX)

S13 Table Mouse visual acuity and contrast sensitivity data. (XLSX)
