# Supplementary material for: APLP2 Regulates Refractive Error and Myopia Development in Mice and Humans
Source: PLoS Genet. 2015 Aug 27;11(8):e1005432. doi: 10.1371/journal.pgen.1005432 (PMC4551475; doi:10.1371/journal.pgen.1005432)
Supplement: S1 Text — (PDF) [file pgen.1005432.s001.pdf]

Supporting Information

APLP2 Regulates Refractive Error and Myopia Development in Mice and Humans

Andrei V. Tkatchenko, Tatiana V. Tkatchenko, Jeremy A. Guggenheim, Virginie J. M. Verhoeven, Pirro G. Hysi, Robert Wojciechowski, Pawan Kumar Singh, Ashok Kumar, Gopal Thinakaran, Consortium for Refractive Error and Myopia (CREAM), Cathy Williams

The Consortium for Refractive Error and Myopia (CREAM) – membership list

Study	Members
1958 British Birth Cohort	Christopher J. Hammond[1], Pirro G. Hysi[1], Jugnoo S. Rahi[2-4]
Aichi cohort	Nagahisa Yoshimura[5], Kenji Yamashiro[5], Masahiro Miyake[5]
ALIENOR	Cécile Delcourt[6,7], Jean-François Korobelnik[6,7]
ALSPAC	Cathy Williams[8], Jeremy A. Guggenheim[9], George McMahon[10], John P. Kemp[10], Beate St Pourcain[10], David M. Evans[10,11], Nicholas J. Timpson[10], George Davey Smith[10]
ANZRAG	Jamie E. Craig[12], Kathryn P. Burdon[12], Rhys D. Fogarty[12]
AREDS1ab	Sudha K. Iyengar[13-15], Robert P. Igo J[13], Emily Chew[16], Sarayut Janmahasatian[13]
AREDS1c	Dwight Stambolian[17], Joan E. Bailey Wilson[18]
BATS	Nicholas G. Martin[19], Yi Lu[20], Stuart MacGregor[20], David Mackey[21,22], Alex Hewitt[21,22]
Beijing Eye Study	Jost B. Jonas[23,24], Ya Xing Wang[24], Liang Xu[24], Seang Mei Saw[25-28]
Blue Mountain Eye Study	Paul N. Baird[21], Maria Schache[21], Paul Mitchell[29], Jie Jin Wang[21,29], Jing Xie[21]
CIEMS	Vinay Nangia[30], Songhomitra Panda-Jonas[30]
CROATIA-Korčula	Caroline Hayward[31], Alan F. Wright[31], Veronique Vitart[31]
CROATIA-Split	Ozren Polasek[32], Harry Campbell[33], Veronique Vitart[31]
CROATIA-Vis	Igor Rudan[33], Zoran Vatavuk[34], Veronique Vitart[31]
CWRU FECD Fuchs Dystrophy	Sudha K. Iyengar[13-15], Robert P. Igo Jr[13], Jeremy R. Fondran[13], Jonathan H. Lass[13,14]
DCCT	Andrew D. Paterson[35], S. Mohsen Hosseini[35]
Duke Myopia Study	Terri L. Young[36], Sheng Feng[36]
EPIC-Norfolk	Robert N. Luben[37], Jing Hua Zhao[38], Anthony P. Khawaja[37], Paul J. Foster[39,40], Kay-Tee Khaw[37], Nick J. Wareham[41]
Estonian Genome Center	Andres Metspalu[42], Toomas Haller[42], Evelin Mihailov[42]
FITSA	Taina Rantanen[43,44], Jaakko Kaprio[45-47],Olavi Pärssinen[43,44,48], Juho Wedenoja[49,50]
Framingham Eye Studies	Joan E. Bailey Wilson[18], Robert Wojciechowski[18,51], Claire L. Simpson[18]
GEMT	Paul N. Baird[21], Maria Schache[21]
Guangzhou High Myopia Registry	Mingguang He[52], Xiaobo Guo[52]
Guangzhou Twin Eye Study	Mingguang He[52], Xiaobo Guo[52]
Gutenberg Health Study	Norbert Pfeiffer[53], René Höhn[53]
Hong Kong cohort study	Chi Pui Pang[54], Li Jia Chen[55], Pancy O. Tam[54], Vishal Jhanji[54,55], Alvin L. Young[54,55]
KORA	Thomas Meitinger[56], Konrad Oexle[56], Janina S. Ried[57], Angela Döring[58,59] and Christian Gieger[57]
Kyoto high myopia	Nagahisa Yoshimura[5], Kenji Yamashiro[5], Masahiro Miyake[5]

LIKI	Olavi Pärssinen[43,44,48]
MESA	Leslie J. Raffel[60], Mary-Frances Cotch[61], Barbara E. Klein[62], Ronald Klein[62], Xiaohui Li[63]
Myopia Genomics Study (Hong Kong HTI)	Shea Ping Yip[64], Maurice K.H. Yap[64]
Nagahama Study	Nagahisa Yoshimura[5], Kenji Yamashiro[5], Masahiro Miyake[5]
Ogliastra Genetic Park Study	Mario Pirastu[65], Federico Murgia[65], Laura Portas[65], Ginevra Biino[66], Maurizio Fossarello[65]
ORCADES	James F. Wilson[33], Brian Fleck[67], Veronique Vitart[31]
Penn Family Studies	Dwight Stambolian[17], Joan E. Bailey Wilson[18]
RAINE	Alex W. Hewitt[21,22], Seyhan Yazar[21,22], David Mackey[21,22], Stuart MacGregor[20], Puya Gharahkhani[20]
Rotterdam Studies (Rotterdam Study I-III, Erasmus Rucphen Family Study, Generation R, MYST)	Virginie J.M. Verhoeven[68,69], Caroline C. Klaver[68,69], Cornelia M. van Duijn[69], Jan Willem L. Tideman[68,69]
Singapore Studies (SCES, SCORM, SIMES, SINDI, SP2, STARS)	Seang-Mei Saw[25-28], Qiao Fan[25], Peng Chen[25], Jiemin Liao[27], Amutha Barathi Veluchamy[26-28], M. Kamran Ikram[25-27], E-Shyong Tai[25,28,70], Tin Aung[26,27], Chiea-Chuen Khor[25,27,71,72], Yik-Ying Teo[25,73], Ching-Yu Cheng[25-27], Tien-Yin Wong[25-27]
TEST	David A. Mackey[21,22], Stuart MacGregor[20], Alex Hewitt[21,22]
TwinsUK & 1958 British Birth Cohort	Christopher J. Hammond[1], Pirro G. Hysi[1], Jugnoo S. Rahi[2-4]
Utah Timorese	Margaret M. Deangelis[74], Margaux Morrison[75], Lindsay Farrer[76]
Wenzhou	Xiangtian Zhou[77], Wei Chen[77]
WESDR	Andrew D. Paterson[35], S. Mohsen Hosseini[35], Barbara E. Klein[62], Ronald Klein[62]
Yokohama Study	Nobuhisa Mizuki[78], Akira Meguro[78]
Young Finns Study	Terho Lehtimäki[79], Kari Matti Mäkelä[79], Olli Raitakari[80,81], Mika Kähönen[82], Ilkka Seppala[79]

CREAM Affiliations

1. Department of Twin Research and Genetic Epidemiology, King's College London School of Medicine, London, UK.
2. Medical Research Council Centre of Epidemiology for Child Health, Institute of Child Health, University College London, London, UK.
3. Institute of Ophthalmology, Moorfields Eye Hospital, London, UK.
4. Ulverscroft Vision Research Group, University College London, London, UK.
5. Department of Ophthalmology and Visual Sciences, Kyoto University Graduate School of Medicine, Kyoto, Japan.
6. Université de Bordeaux, Bordeaux, France.
7. INSERM (Institut National de la Santé Et de la Recherche Médicale), ISPED (Institut de Santé Publique d'Épidémiologie et de Développement), Centre INSERM U897-Epidemiologie-Biostatistique, Bordeaux, France.
8. School of Social and Community Medicine, University of Bristol, Bristol, UK.
9. Centre for Myopia Research, School of Optometry, The Hong Kong Polytechnic University, Hong Kong, Hong Kong.
10. MRC Integrative Epidemiology Unit (IEU), the University of Bristol, Bristol, UK. .
11. University of Queensland Diamantina Institute, Translational Research Institute, Brisbane, Queensland, Australia.
12. Department of Ophthalmology, Flinders University, Adelaide, Australia.
13. Department of Epidemiology and Biostatistics, Case Western Reserve University, Cleveland, Ohio, USA.

14. Department of Ophthalmology and Visual Sciences, Case Western Reserve University and University Hospitals Eye Institute, Cleveland, Ohio, USA.
15. Department of Genetics, Case Western Reserve University, Cleveland, Ohio, USA.
16. National Eye Institute, National Institutes of Health, Bethesda, Maryland, USA.
17. Department of Ophthalmology, University of Pennsylvania, Philadelphia, Pennsylvania, USA.
18. Inherited Disease Research Branch, National Human Genome Research Institute, National Institutes of Health, Baltimore, Maryland, USA.
19. Genetic Epidemiology Laboratory, QIMR Berghofer Medical Research Institute, Herston, Brisbane, Queensland, Australia.
20. Statistical Genetics Laboratory, QIMR Berghofer Medical Research Institute, Herston, Brisbane, Queensland, Australia.
21. Centre for Eye Research Australia (CERA), University of Melbourne, Royal Victorian Eye and Ear Hospital, Melbourne, Victoria, Australia.
22. Centre for Ophthalmology and Visual Science, Lions Eye Institute, University of Western Australia, Perth, Australia.
23. Department of Ophthalmology, Medical Faculty Mannheim, Ruprecht-Karls-University Heidelberg, Mannheim, Germany.
24. Beijing Institute of Ophthalmology, Beijing Tongren Hospital, Capital Medical University, Beijing, China.
25. Saw Swee Hock School of Public Health, National University Health Systems, National University of Singapore, Singapore, Singapore.
26. Singapore Eye Research Institute, Singapore National Eye Centre, Singapore, Singapore.
27. Department of Ophthalmology, National University Health Systems, National University of Singapore, Singapore.
28. DUKE-National University of Singapore Graduate Medical School, Singapore, Singapore.
29. Department of Ophthalmology, Centre for Vision Research, Westmead Millennium Institute, University of Sydney, Sydney, Australia.
30. Suraj Eye Institute, Nagpur, Maharashtra, India.
31. Medical Research Council Human Genetics Unit, Institute of Genetics and Molecular Medicine, University of Edinburgh, Edinburgh, UK.
32. Faculty of Medicine, University of Split, Split, Croatia.
33. Centre for Population Health Sciences, University of Edinburgh, Edinburgh, UK.
34. Department of Ophthalmology, Sisters of Mercy University Hospital, Zagreb, Croatia.
35. Program in Genetics and Genome Biology, Hospital for Sick Children and University of Toronto, Toronto, Ontario, Canada.
36. Department of Pediatric Ophthalmology, Duke Eye Center For Human Genetics, Durham, North Carolina, USA.
37. Department of Public Health and Primary Care, Institute of Public Health, University of Cambridge School of Clinical Medicine, Cambridge, UK. .
38. MRC Epidemiology Unit, Institute of Metabolic Sciences, University of Cambridge, Cambridge, UK.
39. Division of Genetics and Epidemiology, UCL Institute of Ophthalmology, London, UK.
40. NIHR Biomedical Research Centre, Moorfields Eye Hospital NHS Foundation Trust and UCL Institute of Ophthalmology, London, UK.
41. MRC Epidemiology Unit, Institute of Metabolic Science, Addenbrooke's Hospital, Cambridge, UK.
42. Estonian Genome Center, University of Tartu, Tartu, Estonia.
43. Gerontology Research Center and Department of Health Sciences, University of Jyväskylä, Jyväskylä, Finland.
44. Gerontology Research Center, University of Jyväskylä, Jyväskylä, Finland.
45. Department of Public Health, University of Helsinki, Helsinki, Finland.

46. Institute for Molecular Medicine, University of Helsinki, Helsinki, Finland.
47. Department of Mental Health and Alcohol Abuse Services, National Institute for Health and Welfare, Helsinki, Finland.
48. Department of Ophthalmology, Central Hospital of Central Finland, Jyväskylä, Finland.
49. Department of Public Health, Hjelt Institute, University of Helsinki, Finland.
50. Department of Ophthalmology, Helsinki University Central Hospital, Helsinki, Finland.
51. Department of Epidemiology, Johns Hopkins Bloomberg School of Public Health, Baltimore, Maryland, USA.
52. State Key Laboratory of Ophthalmology, Zhongshan Ophthalmic Center, Sun Yat-sen University, Guangzhou, China.
53. Department of Ophthalmology, University Medical Center Mainz, Mainz, Germany.
54. Department of Ophthalmology and Visual Sciences, The Chinese University of Hong Kong Hong Kong Eye Hospital, Kowloon, Hong Kong.
55. Department of Ophthalmology and Visual Sciences, The Chinese University of Hong Kong, Prince of Wales Hospital, Shatin, Hong Kong. .
56. Institute of Human Genetics, Technical University Munich, Munich, Germany.
57. Institute of Genetic Epidemiology, Helmholtz Zentrum München—German Research Center for Environmental Health, Neuherberg, Germany.
58. Institute of Epidemiology I, Helmholtz Zentrum München, German Research Center for Environmental Health, Neuherberg, Germany.
59. Institute of Epidemiology II, Helmholtz Zentrum München, German Research Center for Environmental Health, Neuherberg, Germany.
60. Medical Genetics Institute, Cedars-Sinai Medical Center, Los Angeles, CA, USA.
61. Division of Epidemiology and Clinical Applications, National Eye Institute, Bethesda, Maryland, USA.
62. Department of Ophthalmology and Visual Sciences, University of Wisconsin School of Medicine and Public Health, Madison, WI, USA.
63. Institute for Translational Genomics and Population Sciences, Los Angeles Biomedical Research Institute, Harbor-UCLA Medical Center, United States.
64. Department of Health Technology and Informatics, The Hong Kong Polytechnic University, Hong Kong, Hong Kong. .
65. Institute of Population Genetics, National Research Council, Sassari, Italy.
66. Molecular Genetics, National Research Council, Pavia, Italy.
67. Princess Alexandra Eye Pavilion, Edinburgh, UK.
68. Department of Ophthalmology, Erasmus Medical Center, Rotterdam, The Netherlands.
69. Department of Epidemiology, Erasmus Medical Center, Rotterdam, The Netherlands.
70. Department of Medicine, National University of Singapore, Singapore, Singapore.
71. Department of Pediatrics, National University of Singapore, Singapore, Singapore.
72. Division of Human Genetics, Genome Institute of Singapore, Singapore, Singapore.
73. Department of Statistics and Applied Probability, National University of Singapore, Singapore, Singapore.
74. Department of Ophthalmology and Visual Sciences, University of Utah, Moran Eye Center, Salt Lake City, USA.
75. Department of Ophthalmology and Visual Sciences, John Moran Eye Center, University of Utah, 60 North Mario Capecchi Dr., Salt Lake City, Utah, 84132, USA.
76. Departments of Medicine (Biomedical Genetics), Ophthalmology, Neurology, Epidemiology, and Biostatistics, Boston University Schools of Medicine and Public Health, Boston, USA.
77. Department of Radiology, Southwest Hospital, The Third Military Medical University, Chongqing, China.
78. Department of Ophthalmology, Yokohama City University School of Medicine, Yokohama, Kanagawa, Japan.

79. Department of Clinical Chemistry, Fimlab laboratories and School of Medicine, University of Tampere, Tampere, Finland.
80. Research Centre of Applied and Preventive Medicine, University of Turku, Turku, Finland.
81. Department of Clinical Physiology and Nuclear Medicine, Turku University Hospital, Turku, Finland.
82. Department of Clinical Physiology, Tampere University Hospital and School of Medicine, University of Tampere, Tampere, Finland.
