# Supplementary figures and images for: APLP2 Regulates Refractive Error and Myopia Development in Mice and Humans
Source: PLoS Genet. 2015 Aug 27;11(8):e1005432. doi: 10.1371/journal.pgen.1005432 (PMC4551475; doi:10.1371/journal.pgen.1005432)

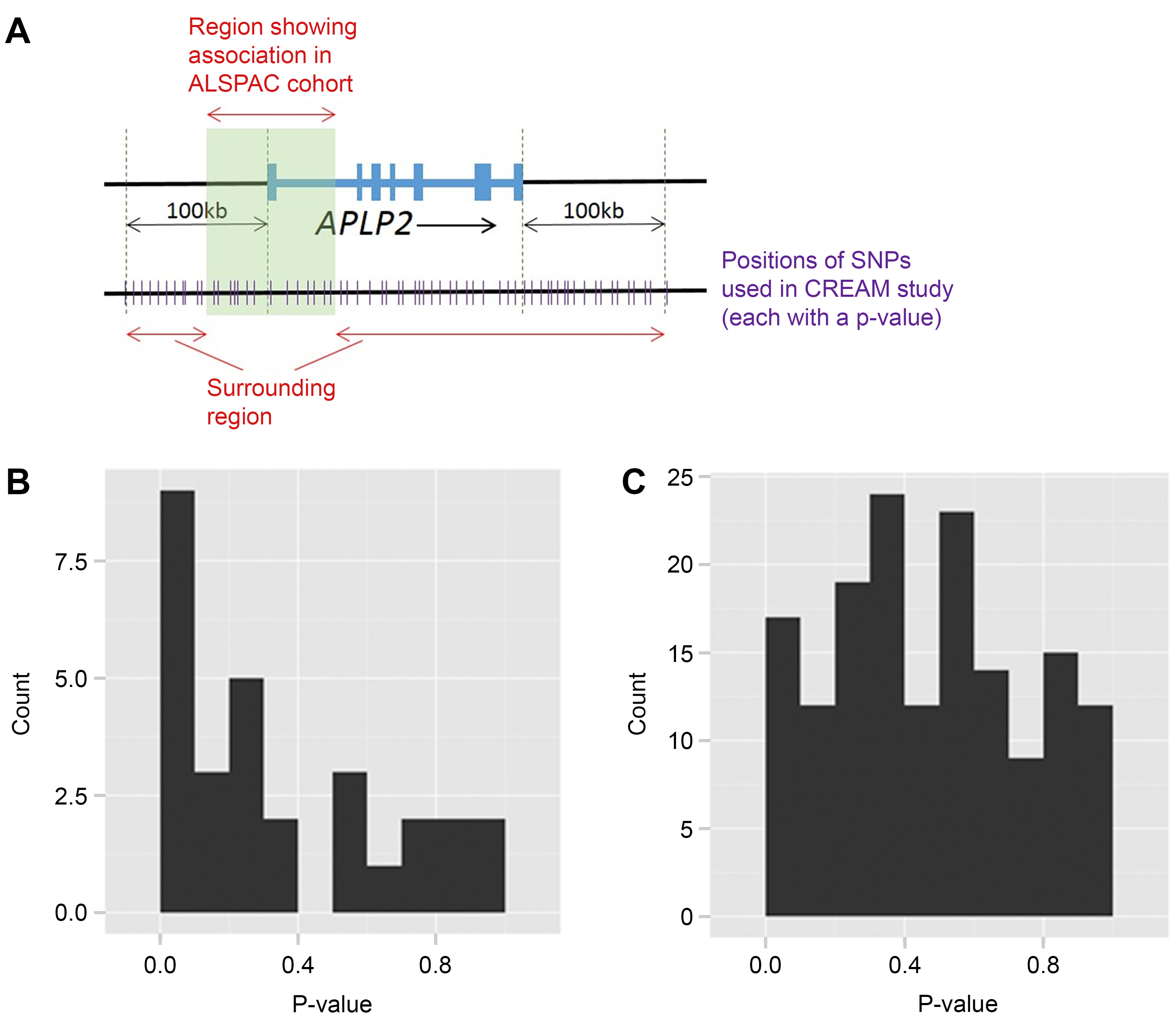

Supplement: S1 Fig — The distribution of p-values was skewed towards unexpectedly low values for SNPs in the region that showed association in ALSPAC participants (hg19 chr 11:129904497–129971498) compared to the surrounding region. The distribution inside the region was significantly skewed towards lower p-values (p = 0.005; two-sample Kolmogorov-Smirnov test) and significantly different from a uniform distribution (p = 0.001; two-sample Kolmogorov-Smirnov test). (A) Schematic diagram of the two regions in which the distribution of p-values was compared: i) the region where a strong association between SNP genotypes and refractive error was observed in the ALSPAC cohort (green shading); and ii) the surrounding region. (B) Distribution of p-values for SNPs within the region showing association in ALSPAC cohort. (C) Distribution of p-values for SNPs in the surrounding region. (TIF) [file pgen.1005432.s002.tif]

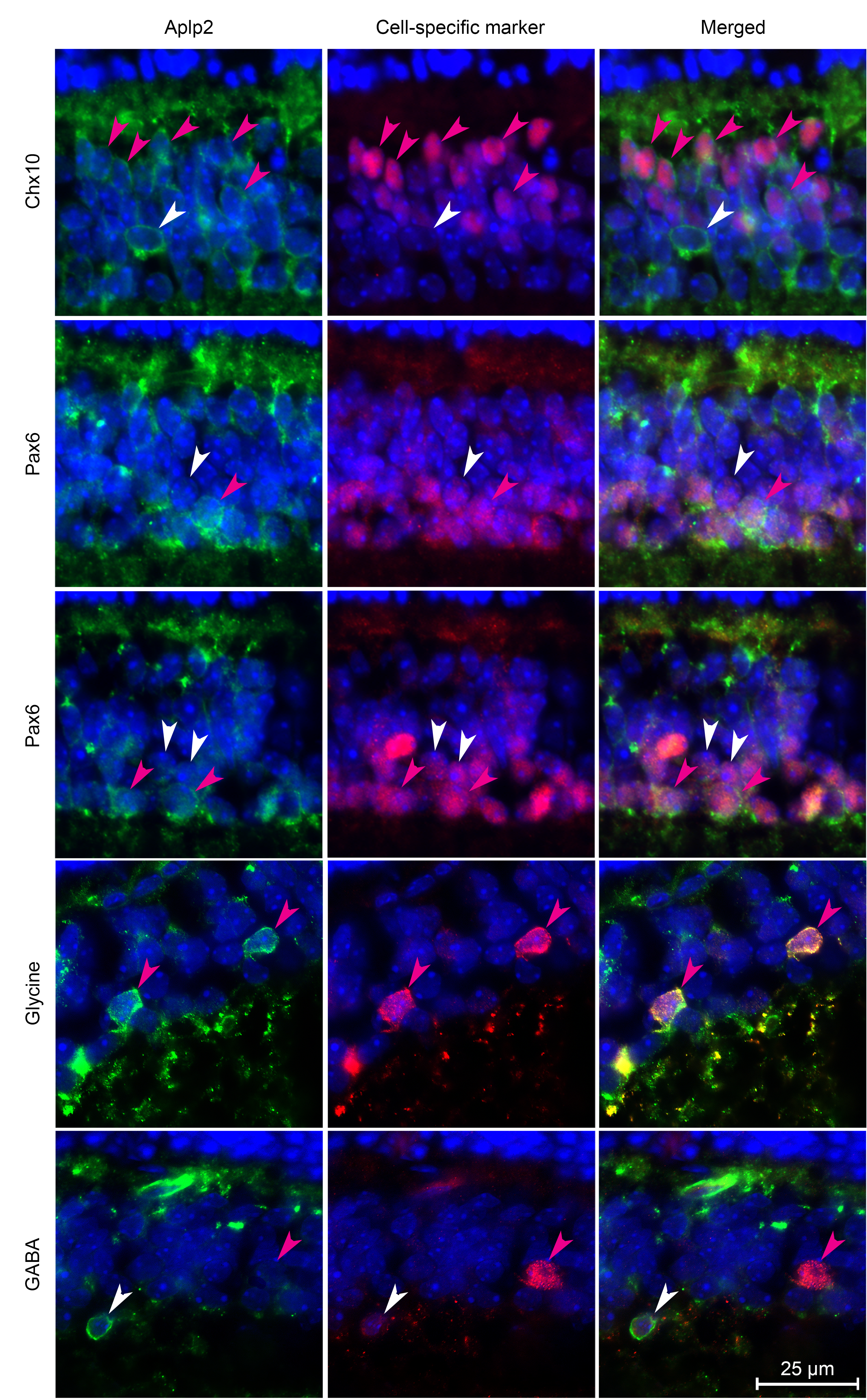

Supplement: S2 Fig — In the inner nuclear layer of the retina, Aplp2 was expressed in the bipolar cells and glycinergic amacrines. (Top panel) Co-localization of Aplp2 and Chx10 demonstrating expression of Aplp2 in the bipolar cells. Magenta arrows, Aplp2- and Chx10-positive bipolar cells; white arrows, Aplp2-positive Chx10-negative cell. (Second and third panels) Co-localization of Aplp2 and Pax6 demonstrating expression of Aplp2 in the amacrine cells. Magenta arrows, Aplp2-positive Pax6-positive amacrine cells; white arrows, Aplp2-negative Pax6-positive amacrine cells. (Fourth panel) Co-localization of Aplp2 and glycine demonstrating expression of Aplp2 in the glycinergic amacrines. Magenta arrows, Aplp2-positive glycine-positive amacrine cells. (Bottom panel) Co-localization of Aplp2 and GABA demonstrating lack of Aplp2 expression in the GABAergic amacrines. Magenta arrows, Aplp2-negative GABA-positive amacrine cell; white arrows, Aplp2-positive GABA-negative (glycinergic) amacrine cell. (TIF) [file pgen.1005432.s003.tif]
