# Supplementary material for: APLP2 Regulates Refractive Error and Myopia Development in Mice and Humans
Source: PLoS Genet. 2015 Aug 27;11(8):e1005432. doi: 10.1371/journal.pgen.1005432 (PMC4551475; doi:10.1371/journal.pgen.1005432)
Supplement: S1 Table — (DOCX) [file pgen.1005432.s004.docx]

**S1 Table. Gene set enrichment analysis – correlation with the depth of the vitreous chamber.**

| Gene symbol | Gene name | Score |
| --- | --- | --- |
| COX11 | Cytochrome c oxidase assembly protein 11 | 2.37 |
| ATP11B | ATPase, Class VI, type 11B | 1.69 |
| ACAD8 | Acyl-Coenzyme A dehydrogenase family, member 8 | 1.55 |
| PRKAR1A | Protein kinase, cAMP-dependent, regulatory, type I, alpha | 1.55 |
| DHX40 | DEAH (Asp-Glu-Ala-His) box polypeptide 40 | 1.24 |
| AY680578 | Macaca mulatta cDNA sl1_h02_t7_455, identical to Homo sapiens clone pDJ181p7 from the Prader-Willi/Angelman Syndrome region | 1.05 |
| AY680579 | Macaca mulatta cDNA sl1_l15_t7_461 | 0.87 |
| PAI-RBP1 | PAI-1 mRNA-binding protein | 0.85 |
| DKFZp434H1419 | Hypothetical protein DKFZp434H1419 | 0.84 |
| PRKWNK1 | Protein kinase, lysine deficient 1 | 0.76 |
| IDH3A | Isocitrate dehydrogenase 3 (NAD+) alpha | 0.70 |
| PODXL | Podocalyxin-like | 0.66 |
| C10orf8 | BUP/PIL protein (chromosome 10 open reading frame 8) | 0.66 |
| TOP2B | DNA topoisomerase II beta | 0.64 |
| CBX3 | Chromobox homolog 3 (HP1 gamma homolog, Drosophila) | 0.63 |
| JIK | STE20-like kinase | 0.60 |
| NLK | Nemo-like kinase | 0.58 |
| DST | Dystonin | 0.46 |
| TEGT | Testis enhanced gene transcript (BAX inhibitor 1) | 0.38 |
| MPRIP | Myosin phosphatase Rho interacting protein | 0.23 |
| ZC3H13 | Zinc finger CCCH-type containing 13 | 0.17 |
| CCDC104 | Coiled-coil domain containing 104 (CCDC104) | 0.12 |
| ZFP91 | Zinc finger protein 91 homolog (mouse) | 0.04 |
| LBH | Likely ortholog of mouse limb-bud and heart gene | 0.03 |
| CLIP3 | CAP-GLY domain containing linker protein 3 (CLIP3) | 0.00 |
| CEP85 | Centrosomal protein 85kDa (CEP85) | 0.00 |
| RBM26 | RNA binding motif protein 26 | -0.01 |
| SCAMP1 | Secretory carrier membrane protein 1 | -0.03 |
| SMN1 | Survival of motor neuron 1 | -0.03 |
| MPEG1 | Macrophage expressed gene 1 | -0.03 |
| ABCD3 | ATP-binding cassette, sub-family D (ALD), member 3 | -0.04 |
| WDR31 | WD repeat domain 31 | -0.04 |
| LRPPRC | Leucine-rich pentatricopeptide repeat containing | -0.04 |
| CALB1 | Calbindin 1, 28kDa | -0.04 |
| PTPRF | Protein tyrosine phosphatase, receptor type, F | -0.05 |
| E2F4 | E2F transcription factor 4, p107/p130-binding | -0.05 |
| IARS | Isoleucine-tRNA synthetase | -0.05 |
| RGS9BP | Regulator of G protein signaling 9 binding protein | -0.07 |
| PCDH9 | Protocadherin 9 | -0.07 |
| VIP | Vasoactive intestinal peptide | -0.08 |
| SNW1 | SNW domain containing 1 | -0.08 |
| SPRYD3 | SPRY domain containing 3 | -0.08 |
| SEC61A1 | Sec61 alpha 1 subunit (S. cerevisiae) | -0.10 |
| CAMSAP1 | Calmodulin regulated spectrin-associated protein 1 | -0.11 |
| NUCKS1 | Nuclear casein kinase and cyclin-dependent kinase substrate 1 | -0.11 |
| MINOS1 | Mitochondrial inner membrane organizing system 1 | -0.12 |
| RPRD2 | Regulation of nuclear pre-mRNA domain containing 2 | -0.13 |
| CCNA2 | Cyclin A2 | -0.15 |
| SMC1L1 | SMC1 structural maintenance of chromosomes 1-like 1 (yeast) | -0.16 |
| KIFAP3 | Kinesin-associated protein 3 | -0.17 |
| GDI2 | GDP dissociation inhibitor 2 | -0.18 |
| DENND4B | DENN/MADD domain containing 4B | -0.18 |
| NCAM1 | Neural cell adhesion molecule 1 | -0.18 |
| PAPOLA | Poly(A) polymerase alpha | -0.18 |
| DDX5 | DEAD (Asp-Glu-Ala-Asp) box polypeptide 5 | -0.18 |
| ANKS1 | Ankyrin repeat and sterile alpha motif domain containing 1 | -0.18 |
| STRC | Stereocilin | -0.18 |
| CNIH | Cornichon homolog (Drosophila) | -0.20 |
| TSR3 | TSR3, 20S rRNA accumulation, homolog (S. cerevisiae) | -0.20 |
| CNOT2 | CCR4-NOT transcription complex, subunit 2 | -0.22 |
| MORF4L2 | Mortality factor 4 like 2 | -0.22 |
| STK40 | Serine/threonine kinase 40 | -0.22 |
| PNUTL2 | Peanut-like 2 (Drosophila) | -0.23 |
| STRADB | STE20-related kinase adaptor beta | -0.23 |
| DGKE | Diacylglycerol kinase, epsilon 64kDa | -0.24 |
| CDC10 | Cell division cycle 10 homolog (S. cerevisiae) | -0.24 |
| RB1 | Retinoblastoma 1 protein | -0.24 |
| CCDC136 | Coiled-coil domain containing 136 | -0.25 |
| MRPL44 | Mitochondrial ribosomal protein L44 | -0.26 |
| GABARAPL1 | GABA(A) receptor-associated protein like 1 | -0.26 |
| ZNF267 | Zinc finger protein 267 | -0.29 |
| hnRNPA3 | Heterogeneous nuclear ribonucleoprotein A3 | -0.31 |
| DDIT4 | DNA-damage-inducible transcript 4 | -0.31 |
| AKAP12 | A kinase (PRKA) anchor protein (gravin) 12 | -0.31 |
| HDGF | Hepatoma-derived growth factor (high-mobility group protein 1-like) | -0.32 |
| AY680531 | Macaca mulatta cDNA ds2_h14_t7_300, identical to Human chromosome 14 DNA sequence BAC R-907D1 | -0.32 |
| BTD | Biotinidase | -0.33 |
| RBBP8 | Retinoblastoma binding protein 8 | -0.35 |
| AY680509 | Macaca mulatta cDNA dl1_m15_t7_232, identical to Human DNA sequence from clone RP11-174C7 on chromosome 6 | -0.36 |
| COX4I1 | Cytochrome c oxidase subunit IV isoform 1 | -0.37 |
| CCNI | Cyclin I | -0.38 |
| SYS1 | SYS1 Golgi-localized integral membrane protein homolog (S. cerevisiae) | -0.39 |
| ANP32A | Acidic (leucine-rich) nuclear phosphoprotein 32 family, member A | -0.39 |
| KCNB1 | Potassium voltage-gated channel, Shab-related subfamily, member 1 | -0.40 |
| CCNB2 | Cyclin B2 | -0.40 |
| FAM18B | Family with sequence similarity 18, member B | -0.41 |
| CREG | Cellular repressor of E1A-stimulated genes | -0.42 |
| JAK1 | Janus kinase 1 (a protein tyrosine kinase) | -0.43 |
| RAX2 | Retina and anterior neural fold homeobox 2 | -0.43 |
| CCNB1 | Cyclin B1 | -0.44 |
| OSBPL2 | Oxysterol binding protein-like 2 | -0.46 |
| SNAP25 | Synaptosomal-associated protein, 25kD | -0.46 |
| LYRM5 | LYR motif containing 5 (LYRM5) | -0.46 |
| BNIP3L | BCL2/adenovirus E1B 19kD-interacting protein 3-like | -0.47 |
| FAM134C | Family with sequence similarity 134, member C | -0.47 |
| ATP6V1F | ATPase, H+ transporting, lysosomal 14kDa, V1 subunit F | -0.49 |
| CNTNAP2 | Contactin associated protein-like 2 | -0.49 |
| ARHGEF12 | Rho guanine nucleotide exchange factor (GEF) 12 | -0.50 |
| NFAT5 | Nuclear factor of activated T-cells 5, tonicity-responsive | -0.51 |
| NTRK2 | Neurotrophic tyrosine kinase, receptor, type 2 | -0.51 |
| ADD3 | Adducin 3 (gamma) | -0.53 |
| ZNF275 | Zinc finger protein 275 | -0.54 |
| ATP5G1 | ATP synthase, H+ transporting, mitochondrial F0 complex, subunit c (subunit 9), isoform 1 | -0.55 |
| SDHC | Succinate dehydrogenase complex, subunit C, integral membrane protein, 15kDa | -0.55 |
| CLU | Clusterin | -0.56 |
| RNF187 | Ring finger protein 187 | -0.58 |
| AY680490 | Macaca mulatta cDNA ds1_i02_t7_159, uncharacterized long non-coding RNA | -0.61 |
| MEP50 | MEP50 protein | -0.62 |
| SPON1 | Spondin 1, (f-spondin) extracellular matrix protein | -0.62 |
| APLP2 | Amyloid beta (A4) precursor-like protein 2 | -0.63 |
| RAB18 | RAB18, member RAS oncogene family | -0.65 |
| XTP3TPB | XTP3-transactivated protein B | -0.73 |
| CUGBP2 | CUG triplet repeat, RNA binding protein 2 | -0.79 |
| PSMB5 | Proteasome (prosome, macropain), subunit beta type 5 | -0.82 |
| OSBPL1A | Oxysterol binding protein-like 1A | -0.83 |
| EPB41L2 | Erythrocyte membrane protein band 4.1-like 2 | -0.90 |
| SNF5 | Transcription factor SNF5 homolog INI1 | -0.94 |
| LGALS8 | Lectin, galactoside-binding, soluble, 8 (galectin 8) | -1.02 |
| RAB10 | RAB10, member RAS oncogene family | -1.28 |
