# Supplementary material for: APLP2 Regulates Refractive Error and Myopia Development in Mice and Humans
Source: PLoS Genet. 2015 Aug 27;11(8):e1005432. doi: 10.1371/journal.pgen.1005432 (PMC4551475; doi:10.1371/journal.pgen.1005432)
Supplement: S2 Table — Model excluding time reading term (n = 5,200). (DOCX) [file pgen.1005432.s005.docx]

**S2 Table. Refractive error “growth trajectory” analysis in ALSPAC subjects. Model excluding time reading term (n = 5,200).**

| **Parameter** | **Beta** | **SE** | **DF** | **t-value** | **P-value** |
| --- | --- | --- | --- | --- | --- |
| rs188663068 (reference = GG) | -3.00 × 10^-02^ | 9.00 × 10^-02^ | 5198 | -3.90 × 10^-01^ | 6.94 × 10^-01^ |
| Age | -27.88 | 6.60 × 10^-01^ | 17434 | -41.96 | < 1.00 ×10^-99^ |
| Age^2^ | -3.78 | 4.00 × 10^-01^ | 17434 | -9.47 | 3.12 × 10^-21^ |
| Age^3^ | 3.27 | 3.80 × 10^-01^ | 17434 | 8.57 | 1.14 × 10^-17^ |
| rs188663068 × Age | -3.00 × 10^-02^ | 1.00 × 10^-02^ | 17434 | -2.69 | 7.00 × 10^-03^ |

SE, standard error of beta coefficient; DF, degrees of freedom.
