# Supplementary material for: APLP2 Regulates Refractive Error and Myopia Development in Mice and Humans
Source: PLoS Genet. 2015 Aug 27;11(8):e1005432. doi: 10.1371/journal.pgen.1005432 (PMC4551475; doi:10.1371/journal.pgen.1005432)
Supplement: S3 Table — Model excluding SNP term (n = 4,461). (DOCX) [file pgen.1005432.s006.docx]

**S3 Table. Refractive error “growth trajectory” analysis in ALSPAC subjects. Model excluding SNP term (n = 4,461).**

| **Parameter** | **Beta** | **SE** | **DF** | **t-value** | **P-value** |
| --- | --- | --- | --- | --- | --- |
| Time reading (reference = “Low”) | -6.00 × 10^-02^ | 3.00 × 10^-02^ | 4459 | -2.03 | 4.30 × 10^-02^ |
| Age | -23.55 | 8.40 × 10^-01^ | 15210 | -28.02 | < 1.00 × 10^-99^ |
| Age^2^ | -3.38 | 4.00 × 10^-01^ | 15210 | -8.54 | 1.50 × 10^-17^ |
| Age^3^ | 2.97 | 3.80 × 10^-01^ | 15210 | 7.82 | 5.61 × 10^-15^ |
| Time reading × Age | -2.00× 10^-02^ | 0.00 | 15210 | -5.77 | 8.26 × 10^-09^ |

SE, standard error of beta coefficient; DF, degrees of freedom.
