# Supplementary material for: APLP2 Regulates Refractive Error and Myopia Development in Mice and Humans
Source: PLoS Genet. 2015 Aug 27;11(8):e1005432. doi: 10.1371/journal.pgen.1005432 (PMC4551475; doi:10.1371/journal.pgen.1005432)
Supplement: S4 Table — Full model (n = 4,461). (DOCX) [file pgen.1005432.s007.docx]

**S4 Table. Refractive error “growth trajectory” analysis in ALSPAC subjects. Full model (n = 4,461).**

| **Parameter** | **Beta** | **SE** | **DF** | **t-value** | **P-value** |
| --- | --- | --- | --- | --- | --- |
| rs188663068 (reference = GG) | 1.00 × 10^-02^ | 1.30 × 10^-01^ | 4457 | 1.20 × 10^-01^ | 9.07 × 10^-01^ |
| Time reading (reference = “Low”) | -5.00 × 10^-02^ | 3.00 × 10^-02^ | 4457 | -1.90 | 5.70 × 10^-02^ |
| Age | -23.56 | 8.50 × 10^-01^ | 15208 | -27.82 | < 1.00 × 10^-99^ |
| Age^2^ | -3.38 | 4.00 × 10^-01^ | 15208 | -8.55 | 1.40 × 10^-17^ |
| Age^3^ | 2.96 | 3.80 × 10^-01^ | 15208 | 7.81 | 6.13 × 10^-15^ |
| rs188663068 × Time reading | -1.2 × 10^-01^ | 1.90 × 10^-01^ | 4457 | -6.50 × 10^-01^ | 5.17 × 10^-01^ |
| rs188663068 × Age | 0.00 | 2.00 × 10^-02^ | 15208 | 7.00 × 10^-02^ | 9.46 × 10^-01^ |
| Time reading × Age | -2.00 × 10^-02^ | 0.00 | 15208 | -5.24 | 1.61 × 10^-07^ |
| rs188663068 × Time reading × Age | -8.00 × 10^-02^ | 3.00 × 10^-02^ | 15208 | -2.84 | 4.00 × 10^-03^ |

SE, standard error of beta coefficient; DF, degrees of freedom.
