# Supplementary material for: APLP2 Regulates Refractive Error and Myopia Development in Mice and Humans
Source: PLoS Genet. 2015 Aug 27;11(8):e1005432. doi: 10.1371/journal.pgen.1005432 (PMC4551475; doi:10.1371/journal.pgen.1005432)
Supplement: S5 Table — Model restricted to time reading “Low” subset (n = 2,775). (DOCX) [file pgen.1005432.s008.docx]

**S5 Table. Refractive error “growth trajectory” analysis in ALSPAC subjects. Model restricted to time reading “Low” subset (n = 2,775).**

| **Parameter** | **Beta** | **SE** | **DF** | **t-value** | **P-value** |
| --- | --- | --- | --- | --- | --- |
| rs188663068 (reference = GG) | 2.00 × 10^-02^ | 1.30 × 10^-01^ | 2773 | 1.40 × 10^-01^ | 8.93 × 10^-01^ |
| Age | -18.46 | 6.10 × 10^-01^ | 9434 | -30.06 | < 1.00 × 10^-99^ |
| Age^2^ | -3.15 | 3.80 × 10^-01^ | 9434 | -8.21 | 2.47 × 10^-16^ |
| Age^3^ | 1.73 | 3.70 × 10^-01^ | 9434 | 4.73 | 2.32 × 10^-06^ |
| rs188663068 × Age | 0.00 | 2.00 × 10^-02^ | 9434 | 2.00 × 10^-02^ | 9.85 × 10^-01^ |

SE, standard error of beta coefficient; DF, degrees of freedom.
