# Supplementary material for: APLP2 Regulates Refractive Error and Myopia Development in Mice and Humans
Source: PLoS Genet. 2015 Aug 27;11(8):e1005432. doi: 10.1371/journal.pgen.1005432 (PMC4551475; doi:10.1371/journal.pgen.1005432)
Supplement: S6 Table — Model restricted to time reading “High” subset (n = 1,686). (DOCX) [file pgen.1005432.s009.docx]

**S6 Table. Refractive error “growth trajectory” analysis in ALSPAC subjects. Model restricted to time reading “High” subset (n = 1,686).**

| **Parameter** | **Beta** | **SE** | **DF** | **t-value** | **P-value** |
| --- | --- | --- | --- | --- | --- |
| rs188663068 (reference = GG) | -1.10 × 10^-01^ | 1.40 × 10^-01^ | 1684 | -7.80 × 10^-01^ | 4.37 × 10^-01^ |
| Age | -19.11 | 7.50 × 10^-01^ | 5772 | -25.57 | < 1.00 × 10^-99^ |
| Age^2^ | -1.43 | 4.10 × 10^-01^ | 5772 | -3.45 | 5.60 × 10^-04^ |
| Age^3^ | 2.57 | 4.00 × 10^-01^ | 5772 | 6.45 | 1.18 × 10^-10^ |
| rs188663068 × Age | -7.00 × 10^-02^ | 2.00 × 10^-02^ | 5772 | -3.39 | 7.12 × 10^-04^ |

SE, standard error of beta coefficient; DF, degrees of freedom.
