# Supplementary material for: APLP2 Regulates Refractive Error and Myopia Development in Mice and Humans
Source: PLoS Genet. 2015 Aug 27;11(8):e1005432. doi: 10.1371/journal.pgen.1005432 (PMC4551475; doi:10.1371/journal.pgen.1005432)
Supplement: S7 Table — Time reading (“Low” versus “High”) (n = 3,312). Without interaction term. (DOCX) [file pgen.1005432.s010.docx]

**S7 Table. Logistic regression model for myopia at age 15½ years in ALSPAC subjects. Time reading (“Low” versus “High”) (n = 3,312). Without interaction term.**

| **Parameter** | **OR** | **L95%** | **U95%** | **P-value** |
| --- | --- | --- | --- | --- |
| Time reading (reference = "Low") | 1.61 | 1.33 | 1.93 | 6.44 × 10^-07^ |
| rs188663068 (reference = GG) | 1.98 | 1.02 | 3.87 | 4.46 × 10^-02^ |

OR, odds ratio; L95%, lower 95% confidence interval; U95%, upper 95% confidence interval.
