# Supplementary material for: APLP2 Regulates Refractive Error and Myopia Development in Mice and Humans
Source: PLoS Genet. 2015 Aug 27;11(8):e1005432. doi: 10.1371/journal.pgen.1005432 (PMC4551475; doi:10.1371/journal.pgen.1005432)
Supplement: S8 Table — Time reading (“Low” versus “High”) (n = 3,312). With interaction term. (DOCX) [file pgen.1005432.s011.docx]

**S8 Table. Logistic regression model for myopia at age 15½ years in ALSPAC subjects. Time reading (“Low” versus “High”) (n = 3,312). With interaction term.**

| **Parameter** | **OR** | **L95%** | **U95%** | **P-value** |
| --- | --- | --- | --- | --- |
| Time reading (reference = "Low") | 1.54 | 1.28 | 1.86 | 7.75 × 10^-06^ |
| rs188663068 (reference = GG) | 0.71 | 0.20 | 2.57 | 5.99 × 10^-01^ |
| rs188663068 × Time reading | 5.42 | 1.15 | 25.52 | 3.26 × 10^-02^ |

OR, odds ratio; L95%, lower 95% confidence interval; U95%, upper 95% confidence interval.
