# Supplementary material for: APLP2 Regulates Refractive Error and Myopia Development in Mice and Humans
Source: PLoS Genet. 2015 Aug 27;11(8):e1005432. doi: 10.1371/journal.pgen.1005432 (PMC4551475; doi:10.1371/journal.pgen.1005432)
Supplement: S9 Table — Time reading (“Low” versus “High”) (n = 3,312). (DOCX) [file pgen.1005432.s012.docx]

**S9 Table. Linear regression model for refractive error at age 15½ years in ALSPAC subjects. Time reading (“Low” versus “High”) (n = 3,312).**

| **Parameter** | **Beta** | **L95%** | **U95%** | **P-value** |
| --- | --- | --- | --- | --- |
| Intercept | -3.10 × 10^-01^ | -3.60 × 10^-01^ | -2.50 × 10^-01^ | 2.10 × 10^-25^ |
| Time reading (reference = "Low") | -2.00 × 10^-01^ | -2.90 × 10^-01^ | -1.00 × 10^-01^ | 2.70 × 10^-05^ |
| rs188663068 (reference = GG) | -9.00 × 10^-02^ | -5.40 × 10^-01^ | 3.70 × 10^-01^ | 7.10 × 10^-01^ |
| Time reading × rs188663068 | -7.40 × 10^-01^ | -1.39 × 10^-01^ | -9.00 × 10^-02^ | 2.70 × 10^-02^ |

L95%, lower 95% confidence interval; U95%, upper 95% confidence interval.
