# Supplementary material for: APLP2 Regulates Refractive Error and Myopia Development in Mice and Humans
Source: PLoS Genet. 2015 Aug 27;11(8):e1005432. doi: 10.1371/journal.pgen.1005432 (PMC4551475; doi:10.1371/journal.pgen.1005432)
Supplement: S10 Table — Time reading (“Low” versus “High”) (n = 3,312). (DOCX) [file pgen.1005432.s013.docx]

**S10 Table. Linear regression model for refractive error at age 15½ years in ALSPAC subjects. Time reading (“Low” versus “High”) (n = 3,312).**

| **Parameter** | **Beta** | **L95%** | **U95%** | **P-value** |
| --- | --- | --- | --- | --- |
| Intercept | -3.00 × 10^-01^ | -3.60 × 10^-01^ | -2.40 × 10^-01^ | 1.10 × 10^-24^ |
| Time reading (reference = "Low") | -2.10 × 10^-01^ | -3.00 × 10^-01^ | -1.20 × 10^-01^ | 5.40 × 10^-06^ |
| rs188663068 (reference = GG) | -4.40 × 10^-01^ | -7.70 × 10^-01^ | -1.20 × 10^-01^ | 7.80 × 10^-03^ |

L95%, lower 95% confidence interval; U95%, upper 95% confidence interval.
