# Supplementary material for: APLP2 Regulates Refractive Error and Myopia Development in Mice and Humans
Source: PLoS Genet. 2015 Aug 27;11(8):e1005432. doi: 10.1371/journal.pgen.1005432 (PMC4551475; doi:10.1371/journal.pgen.1005432)
Supplement: S11 Table — Time outdoors (“Low” versus “High”) (n = 3,329). (DOCX) [file pgen.1005432.s014.docx]

**S11 Table. Linear regression model for refractive error at age 15½ years in ALSPAC subjects. Time outdoors (“Low” versus “High”) (n = 3,329).**

| **Parameter** | **Beta** | **L95%** | **U95%** | **P-value** |
| --- | --- | --- | --- | --- |
| Intercept | -4.50 × 10^-01^ | -5.10 × 10^-01^ | -3.90 × 10^-01^ | 1.20 × 10^-46^ |
| Time outdoors (reference = "Low") | 1.50 × 10^-01^ | 6.00 × 10^-02^ | 2.30 × 10^-01^ | 1.50 × 10^-03^ |
| rs188663068 (reference = GG) | -3.00 × 10^-01^ | -7.30 × 10^-01^ | 1.20 × 10^-01^ | 1.60 × 10^-01^ |
| Time outdoors × rs188663068 | -3.60 × 10^-01^ | -1.02 | 3.00 × 10^-01^ | 2.80 × 10^-01^ |

L95%, lower 95% confidence interval; U95%, upper 95% confidence interval.
